# A nano-innate immune system activator for cancer therapy in a 4T1 tumor-bearing mouse model

**DOI:** 10.1186/s12951-022-01265-4

**Published:** 2022-01-29

**Authors:** Xiang-Yu Liu, Mao-Hua Zhu, Xiao-Yu Wang, Xiao Dong, Hai-Jun Liu, Rui-Yang Li, Shi-Chong Jia, Qin Lu, Mei Zhao, Peng Sun, Hong-Zhuan Chen, Chao Fang

**Affiliations:** 1 Hongqiao International Institute of Medicine, Tongren Hospital and State Key Laboratory of Oncogenes and Related Genes, Department of Pharmacology and Chemical Biology, Shanghai Jiao Tong University School of Medicine (SJTU-SM), Shanghai, 200025 China; 2 Department of Pharmacy, Shanghai University of Medicine and Health Sciences, 279 Zhouzhu Road, Shanghai, 201318 China; 3 Department of General Surgery, Tongren Hospital, SJTU-SM, Shanghai, 200336 China; 4 Institute of Interdisciplinary Integrative Biomedical Research, Shuguang Hospital, Shanghai University of Traditional Chinese Medicine, Shanghai, 201203 China; 5 Key Laboratory of Basic Pharmacology of Ministry of Education & Joint International Research Laboratory of Ethnomedicine of Ministry of Education, Zunyi Medical University, Zunyi, 563003 China

**Keywords:** Nanoparticles, Innate immune system, Fc fragment, Immunotherapy, Breast cancer

## Abstract

**Background:**

Harnessing the immune system to fight cancer has led to prominent clinical successes. Strategies to stimulate innate immune effectors are attracting considerable interest in cancer therapy. Here, through conjugating multivalent Fc fragments onto the surface of mesoporous silica nanoparticles (MSN), we developed a nanoparticle-based innate immune system activator (NISA) for breast cancer immunotherapy.

**Methods:**

NISA was prepared through conjugating mouse IgG3 Fc to MSN surface. Then, long-chain PEG_5000_, which was used to shield Fc to confer nanoparticle colloidal stability, was linked to the MSN surface via matrix metalloprotease-2 (MMP-2)-cleavable peptide (GPLGIAGQC). The activation of multiple components of innate immune system, including complement and the innate cells (macrophages and dendritic cells) and the associated anticancer effect were investigated.

**Results:**

Fc fragments of NISA can be exposed through hydrolysis of long-chain PEG_5000_ by highly expressed MMP-2 in tumor microenvironment. Then, effective stimulation and activation of multiple components of innate immune system, including complement, macrophages, and dendritic cells were obtained, leading to efficient antitumor effect in 4T1 breast cancer cells and orthotopic breast tumor model in mice.

**Conclusions:**

The antitumor potency conferred by NISA highlights the significance of stimulating multiple innate immune elements in cancer immunotherapy.

**Graphical Abstract:**

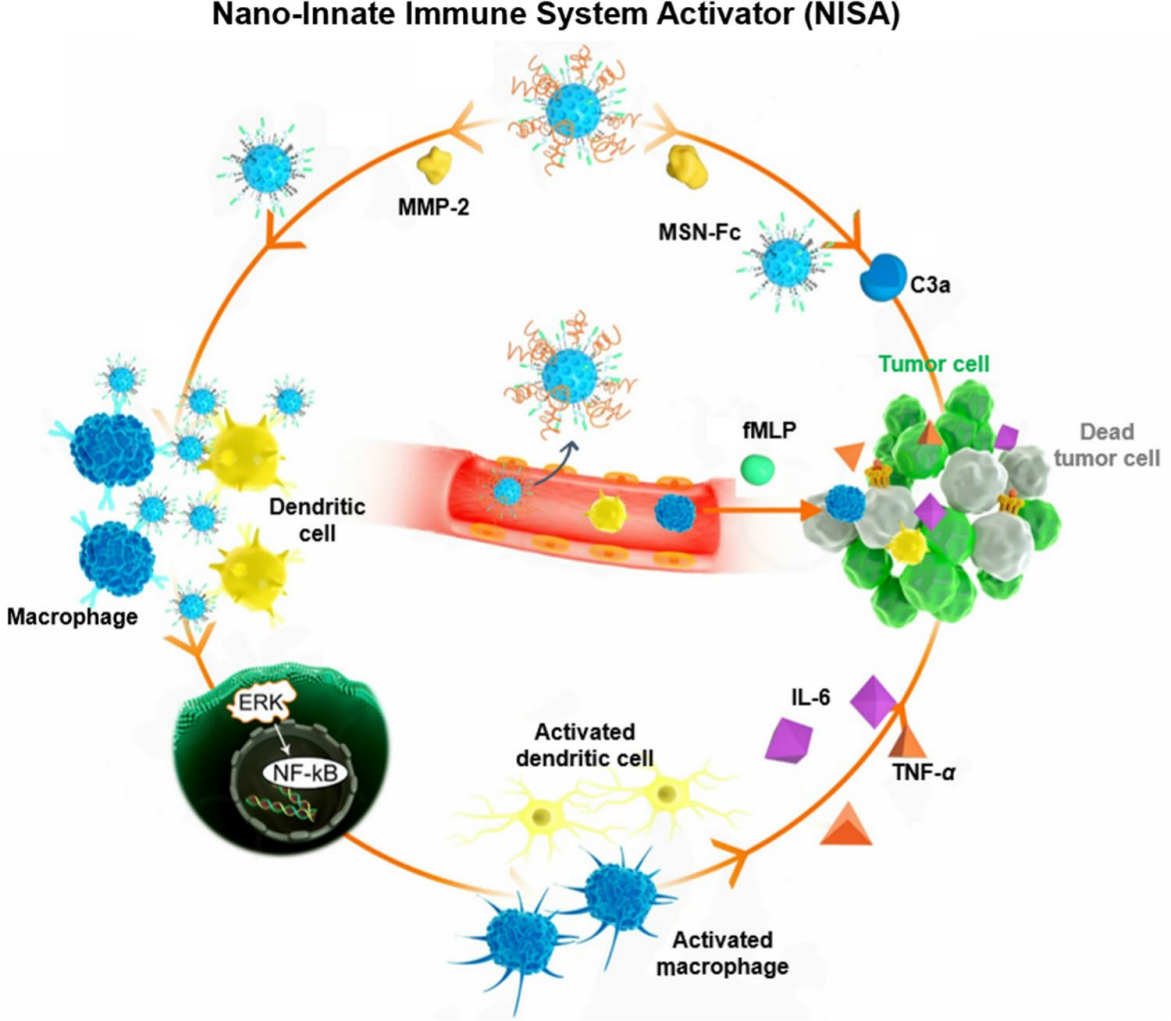

**Supplementary Information:**

The online version contains supplementary material available at 10.1186/s12951-022-01265-4.

## Introduction

The recent clinical successes of cancer immunotherapy (PD-1/PD-L1 immune checkpoint blockade and chimeric antigen receptor T cell therapy) are attracting considerable interest in harnessing the immune system to fight cancer [1–4]. To date, many nanoparticle-based strategies have been explored to enhance the efficacy of cancer immunotherapy. The nanoparticles can either directly exhibit intrinsic immunomodulatory properties to boost antitumor immune responses or be mostly used as a platform to deliver immunostimulatory agents to various targeted cells for immunotherapy [5–13].

Nanoparticle-mediated delivery of tumor-associated antigens and immune adjuvant to enhance the CD8^+^ cytotoxic T lymphocyte responses is the most common strategy [14, 15]. Besides, increasing efforts of using nanoparticles to strengthen innate immune is emerging as a new strategy in cancer immunotherapy. The main players of innate immunity system relative to cancer immunotherapy includes complement system and innate immune cells. Activated complement system produces membrane attack complexes (MACs) to attack tumor cells and the generated anaphylatoxins (C3a and C5a) can also recruit leukocytes to tumor sites [16]. Innate immune cells can impart antitumor immunity through multiple indirect and direct way. Macrophages polarized toward the M1 phenotype secrete nitric oxide (NO) species to activate endothelial cells and chemokines and promote the recruitment of T cells to tumor sites [17]. Natural killer (NK) cells are able to recognize the specific molecules (glycolipids and stress ligands) expressed by tumor cells, leading to innate cell activation and tumor cell lysis [18]. Dendritic cells (DCs) process and present tumor antigens to T cells, which builds a bridge between innate and adaptive immune system [19]. The innate immune effectors can also shape the tumor microenvironment to affect DC activation and differentiation of effector T cells, and augment T-cell recruitment to tumors [20, 21]. It showed that co-delivery of TGF-*β* inhibitor using nanoparticles increased the activity of NK cells in tumors, leading to an extended survival of tumor-bearing mice [22]. Iron oxide nanoparticles inhibited tumor growth by inducing pro-inflammatory macrophage polarization in tumor tissues [23]. TLR7/8-agonist-loaded nanoparticles promoted the polarization of tumor-associated macrophages for enhanced cancer immunotherapy [24]. However, to our knowledge, there have been fewer reports on nanoparticles that can simultaneously target multiple components of innate immune system.

**Scheme 1:**
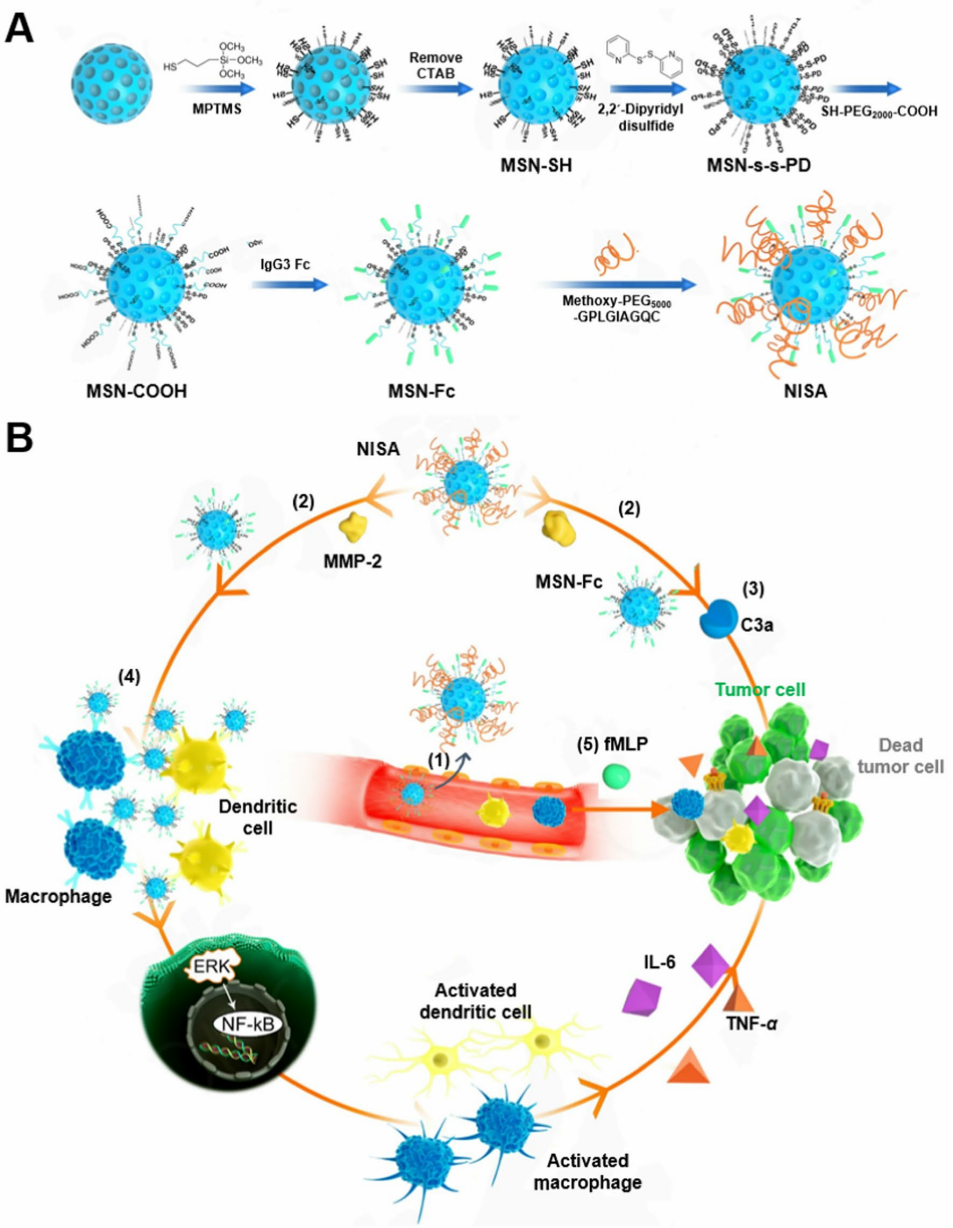
Schematic illustration of the preparation (**A**) and proposed mechanism (**B**) of NISA. (1) NISA accumulated in tumor site via EPR effect after i.v. injection. (2) NISA detaches the long-chain PEG_5000_ to expose the Fc (MSN-Fc) in the presence of MMP-2 in tumor microenvironment. (3) MSN-Fc activate the complement system (C3a) to attack tumor cells. (4) Pro-inflammatory stimulation of innate immune cells (macrophages and dendritic cells) by MSN-Fc. (5) fMLP helps recruit more innate immune cells to tumor sites, where they are stimulated by NISA for enhanced cancer therapy

For this purpose, we developed a nano-innate immune system activator (NISA), using the mesoporous silica nanoparticles (MSN) as the scaffold, for immunotherapy in a mouse 4T1 breast cancer model. NISA was engineered through conjugating mouse IgG3 Fc to MSN surface. IgG3 generally has the strongest binding capacity to Fc receptors expressed on innate immune cells (macrophages and DCs) among all IgG subclasses. This binding can drive the production and release of pro-inflammatory cytokines and chemokines [25, 26]. Fc portion of IgG3 also efficiently triggers the complement cascade through binding C1q [27–29]. Complement activation leads to the formation of membrane attack complex (MAC), which would cause cancer cell death [30, 31]. Then, long-chain PEG_5000_, which was used to shield Fc to confer nanoparticle colloidal stability, was linked to the MSN surface via matrix metalloprotease-2 (MMP-2)-cleavable peptide (GPLGIAGQC) [32] (Scheme 1A). After nanoparticle accumulation in tumors via the enhanced permeation and retention (EPR) effect, the MMP2-cleavable peptide was hydrolyzed by highly expressed MMP-2 in tumor microenvironment, then Fc fragments were exposed to activate the multiple innate immune components (complement, macrophages and dendritic cells) to attack tumor (Scheme 1B). In this study, MSN was used as the scaffold to conjugate IgG3 Fc fragments on the particle surface. This design conferred a multivalent Fc display for effective complement activation which requires separation of Fc domains by no more than ~ 40 nm [33]. The binding sites for complement C1q (the initial molecule in complement cascade) and Fcγ receptors are partially or completely shielded by Fab arms [27], thus the use of non-immune control IgG3 is expected to be ineffective. Further, when i.v. administered, raw Fc fragments would be quickly cleared by mononuclear phagocytes due to the direct exposure of the binding sites. NISA can delay the growth of orthotopic 4T1 breast cancer in mouse, and the effect would be further improved in the presence of *N*-formyl-methionyl-leucyl-phenylalanine (fMLP), a classical chemotactic peptide for the recruitment of immune cells into sites of inflammation, such as solid tumors for antitumor immunotherapy [34–36]. The physicochemical properties of NISA are characterized and the anticancer effect through stimulation and activation of multiple innate immune components in vitro and in vivo demonstrated.

## Results and discussion

### Preparation and characterization of NISA

**Fig. 1:**
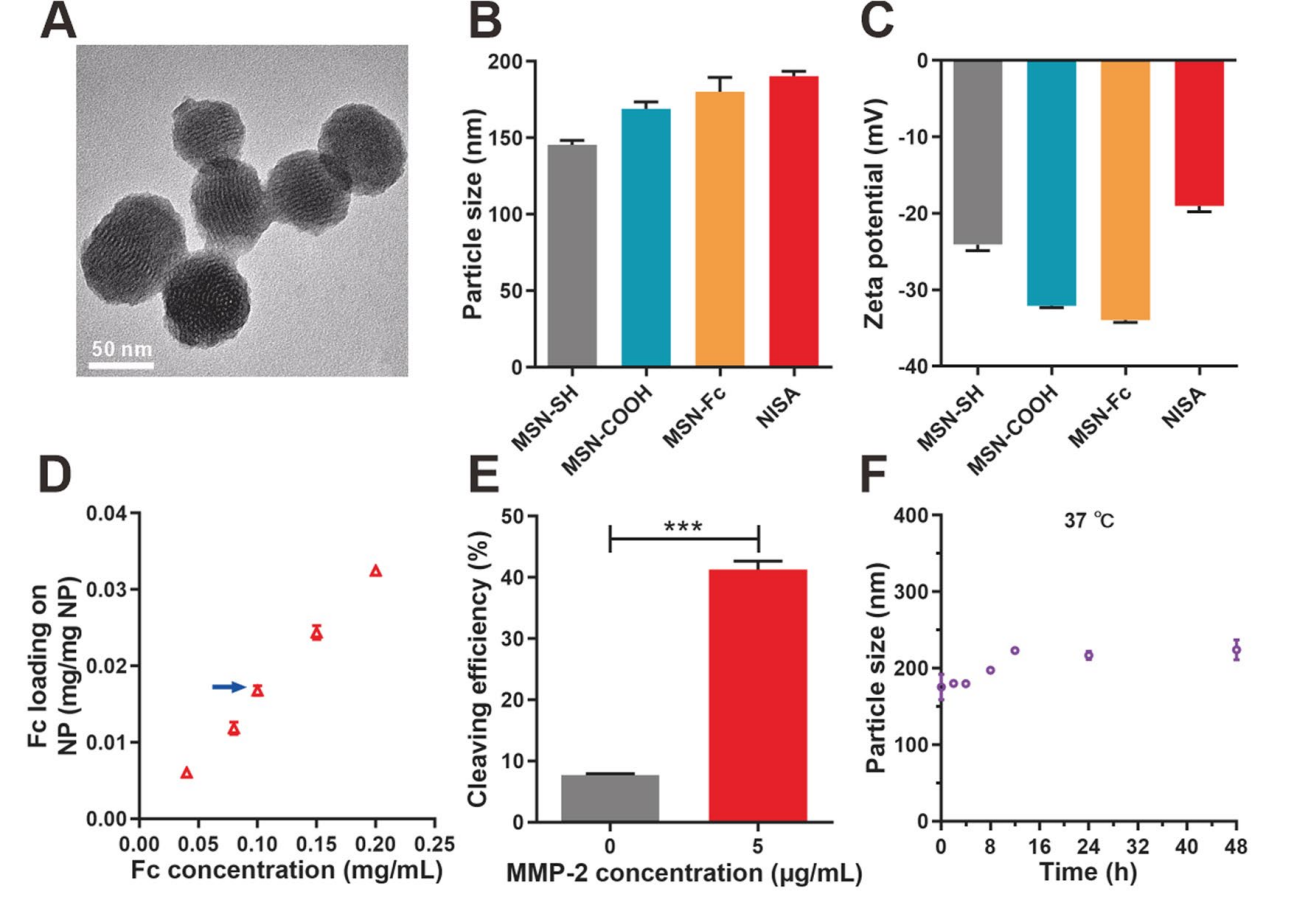
Characterization of NISA. **A** TEM photograph. Bar, 50 nm. **B** Size determined by dynamic light scattering. **C** Zeta potential. **D** Fc loading on the nanoparticles (NP). The intermediate Fc loading formulation indicated with an arrow was adopted for final NISA generation. **E** Cleaving efficiency of MMP-2 sensitive peptide. **F** Colloid stability of NISA in PBS with 10% FBS at 37 ℃. Data are presented as mean ± SD (n = 3). ***p < 0.001

NISA were spherical with rough surface, compared to the nude MSN (Additional file 1: Fig. S1), in the image of transmission electron microscopy (TEM), which may be due to the coverage with Fc fragments and PEG (Fig. 1A). The evolution of size and zeta potential during NISA preparation reflected the sequential surface modification and conjugation of Fc fragment and PEG molecules (Fig. 1B, C, Additional file 1: Fig. S2). To confirm the completion of each synthesis step, we further performed X-ray photoelectron spectroscopy (XPS) test to identify the surface element components of the nanoparticles (Additional file 1: Fig. S3) [37]. N1s with extremely low content (0.40%) in MSN-SH was from the pore-generating template (CTAB) left on the particle surface. Functionalization with 2,2′-dipyridyldisulfide conferred increased surfaced N1s (1.68%) of MSN-s-s-PD. After partial PEGylation (MSN-COOH), surface N1s declined to 0.92%. Lastly, IgG3 Fc conjugation (MSN-Fc) led to dramatically elevated N1s to 3.18%. The changes of surface N1s confirmed the sequential modification and loading of the functional molecules and IgG3 Fc.

Fc fragments can be efficiently linked onto the particle surface with above 90% conjugation efficiency in the tested concentration range (Additional file 1: Fig. S4). This was also proved by SDS-PAGE assay (Additional file 1: Fig. S5). We then chose the intermediate Fc loading formulation (0.0168 mg Fc/mg nanoparticles, indicated with an asterisk in Fig. 1D) for final NISA preparation in this study. We further determined the number of Fc on each nanoparticle according to the method previously described [38, 39]. The weight of each nanoparticle (m) was calculated using the formula: m = ρ*(πD^3^/6). ρ was the nanoparticle density estimated to be 1 g/cm^3^, and D was the number-based mean particle diameter (190 nm) determined by DLS. Thus, the number of Fc on each nanoparticle was estimated to be 1400. 40% of the long-chain PEG_5000_ can be hydrolyzed by MMP-2 at 5 µg/mL (0.07 µM), a concentration 90% lower than that in the tumor microenvironment (1 µM) [40], after 12 h incubation (Fig. 1E). This relatively high sensitivity would be helpful to overcome the limitation caused by the tumor heterogeneity. Thus, the Fc fragments on the particle surface are expected to be efficiently exposed and activate the complement as well as the innate immune cells through interaction with Fc receptors on the cell surface. Moreover, IgG3 Fc remained stable on the nanoparticle surface with no observable shedding (Additional file 1: Fig. S6), ensuring a multivalent Fc display for efficient complement activation and signaling activation of immune cells. The size of NISA maintained well in PBS with 10% FBS at 37 ℃ and in PBS at 4 ℃ (Fig. 1F, Additional file 1: Fig. S7), indicating the good colloid stability in both physiological environment and storage condition.

### Pro-inflammatory stimulation of macrophages and dendritic cells

**Fig. 2:**
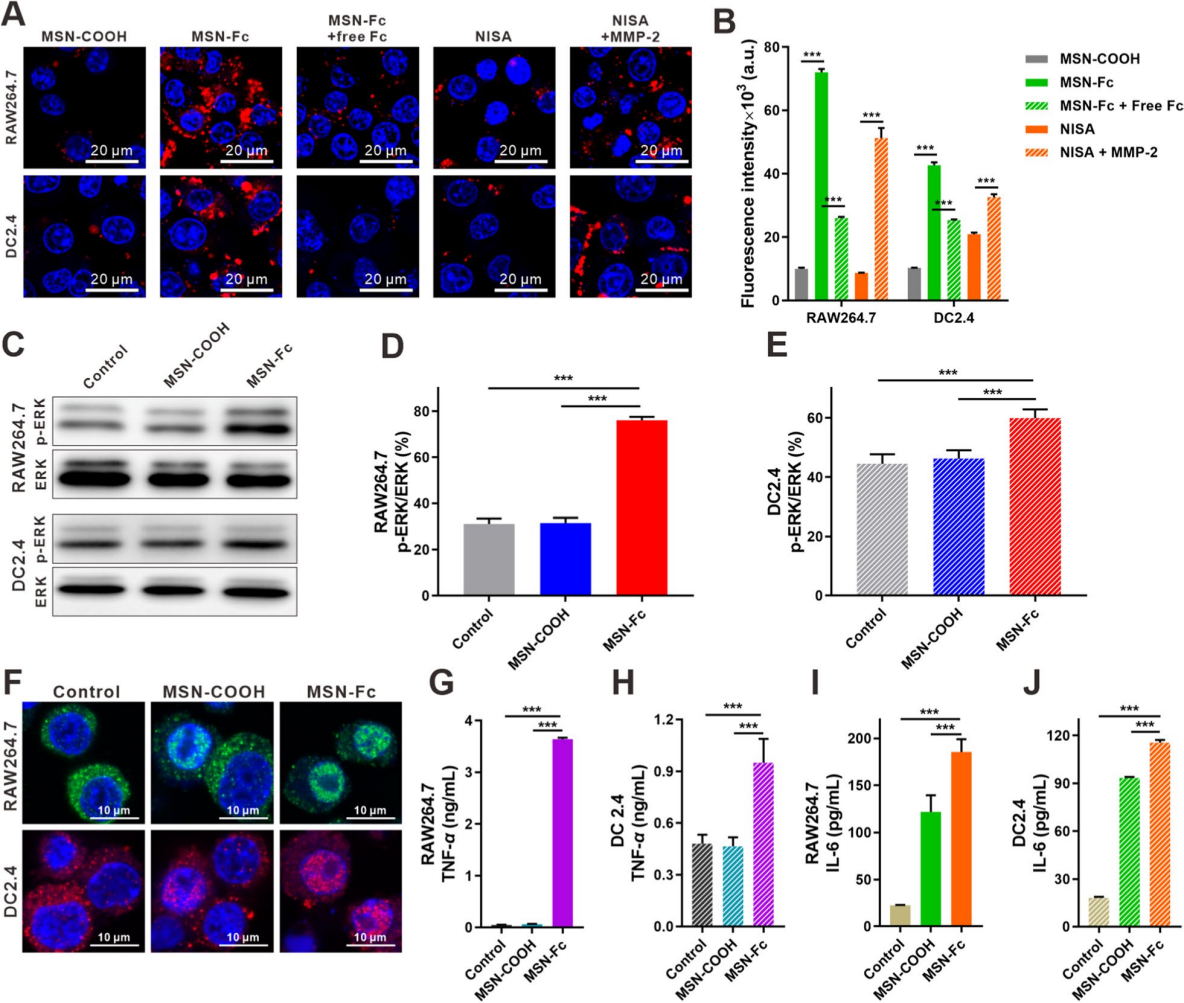
Fc on the nanoparticle surface induced pro-inflammatory stimulation of macrophages and dendritic cells. **A** Binding of the nanoparticles to RAW264.7 and DC2.4 cells through Fc and Fcγ interaction. MSN particles (red) were labeled with iFluor 647. **B** Statistical assay of the cell-associated nanoparticle fluorescence intensity. Western blot (**C**) and statistical assay (**D**, **E**) of p-ERK expression in both cells. **F** Identification of NF-κB intracellular location. NF-κB was stained with Alexa Fluor 488 (for RAW264.7 assay, green) or iFluor 647 (for DC2.4 assay, red) fluorescent antibody, respectively. The secretion of TNF-*α* (**G**, **H**) and IL-6 (**I**, **J**) from both cells were determined using Elisa kit. Data are presented as mean ± SD (n = 3). ***p < 0.001

Fc displayed on the nanoparticle surface is expected to be recognized by the specific Fcγ receptors on the membrane of leukocytes (macrophages and dendritic cells) (Additional file 1: Fig. S8), leading to the pro-inflammatory stimulation of effector cells and the production of cytotoxic cytokines. Compared to MSN-COOH, exposed Fc fragments mediated more binding of MSN-Fc to both macrophages (RAW264.7) and dendritic cells (DC2.4), and this role can be dramatically blocked by free Fc (Fig. 2A, B). Long-chain PEG_5000_ attenuated the binding of NISA on RAW264.7 and DC2.4 cells. However, pre-treatment with MMP-2 (NISA + MMP-2) dramatically enhanced the binding through exposing Fc via the hydrolytic cleavage of the peptide linker (Fig. 2A, B). This observation is consistent with the expectation that in the circulation Fc on the particles can be protected and exert the role after transportation to the tumor site. Moreover, the binding of MSN-Fc stimulated the activation of ERK with the increased phosphorylation, compared to that of control and nanoparticles without Fc (MSN-COOH) (Fig. 2C–E). ERK activation in immune cells was also compared between the treatments with free Fc and MSN-Fc (Additional file 1: Fig. S9). MSN-Fc stimulated more ERK activation in both RAW264.7 and DC2.4 cells, compared to free Fc. This observation is consistent with the finding that clustered antibody fragments can effectively enhance signaling [41]. ERK activation triggered the rapid translocation of NF-kB into the nuclei, which was ready for cytokine production (Fig. 2F). MSN-Fc treatment generated above 60-fold more secretion of TNF-*α* from RAW264.7 and ~ 2-fold from DC2.4, compared to control and MSN-COOH (Fig. 2G, H). IL-6 secretions were also obviously increased in both cells treated with MSN-Fc (Fig. 2I, J). The improved secretion of cytokines indicated the activation of the immune cells. Moreover, the pseudopodium of the cells was observed through the immunofluorescent staining of F-actin. MSN-Fc treatment induced the formation of more pseudopodiums in macrophages and DCs (Additional file 1: Fig. S10), which are required for the innate immune cells to move and exert their action.

### Complement activation triggered by MSN-Fc and its effect on 4T1 cell viability

**Fig. 3:**
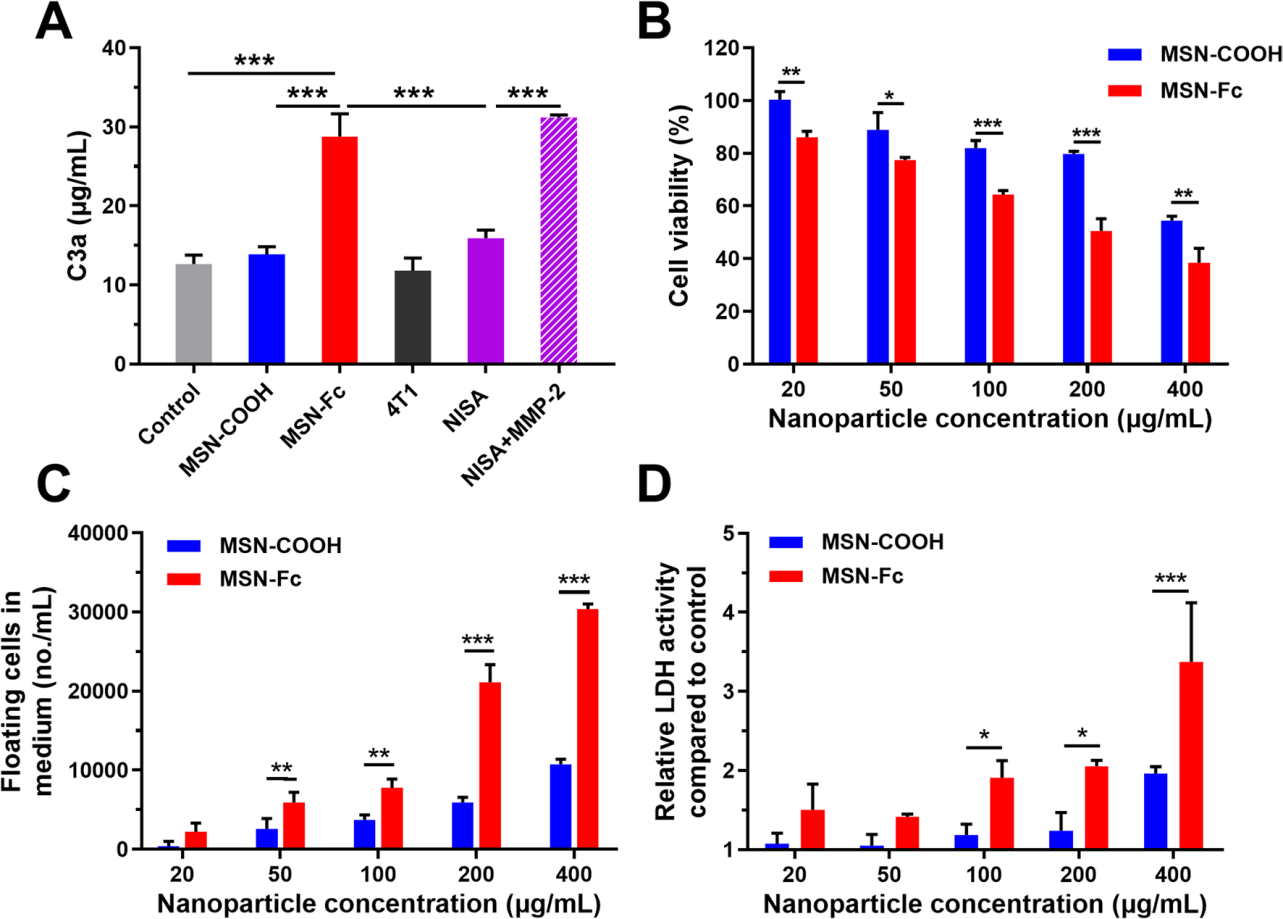
Complement activation and its effect on 4T1 cell viability. **A** C3a generation after nanoparticle incubation with fresh guinea pig serum. For NISA+MMP-2 treatment, NISA was previously incubated with MMP-2 (5 µg/mL) for 12 h at 37 ℃. 4T1 cell alone was also used as a control. **B** 4T1 cell viabilities after MSN-Fc treatment were determined using CCK-8 assay. **C** Floating dead or unhealthy cells in the supernatant medium were counted after nanoparticle (20–400 µg/mL) treatment. **D** Relative LDH activity was assayed after nanoparticle treatment. Data are presented as mean ± SD (n = 3). *p < 0.05, **p < 0.01, and ***p < 0.001

C3a is the representative anaphylatoxin generated in the process of complement activation [16]. We detected the C3a content in the fresh guinea pig serum incubated with nanoparticles. It showed that MSN-Fc induced twofold higher C3a production compared to MSN-COOH and control (Fig. 3A), indicating that the Fc fragments on the particles can effectively activate the complement system. With the long-chain PEG_5000_ protection, the ability of NISA in activating complement was largely suppressed. However, after MMP-2 pre-treatment, C3a activation was recovered to the level comparable to that of MSN-Fc. Notably, the serum MMP-2 levels of breast cancer patients were reported to be ~ 24 ng/mL [42], 200-folds lower than that (5 µg/mL) used in the in vitro test (Fig. 1E). Thus, with the shielding of the long-chain PEG_5000_, NISA-induced complement activation in serum would be very limited, ensuring good safety. 4T1 cell alone cannot induce more C3a production compared to control and MSN-COOH (Fig. 3A).The activation of complement system may lead to the formation of membrane attack complex (MAC), which can disrupt the phospholipid bilayer of the cell membrane, leading to cell lysis and death [16]. MSN-Fc significantly decreased the cell viability compared to MSN-COOH at all tested concentrations (Fig. 3B). Consistently, more floating dead or unhealthy cells in the supernatant medium were observed for the treatment with MSN-Fc (Fig. 3C). Detection of LDH release is a common method to evaluate cytotoxicity [43]. LDH activities in the medium for the cells treated with MSN-Fc were much higher than those treated with MSN-COOH (Fig. 3D). These observations suggested that compared to MSN-COOH, MSN-Fc were more toxic to 4T1 cells, which would be partially caused by the Fc-triggered complement activation.

### Blood clearance and in vivo tumor targeting

**Fig. 4:**
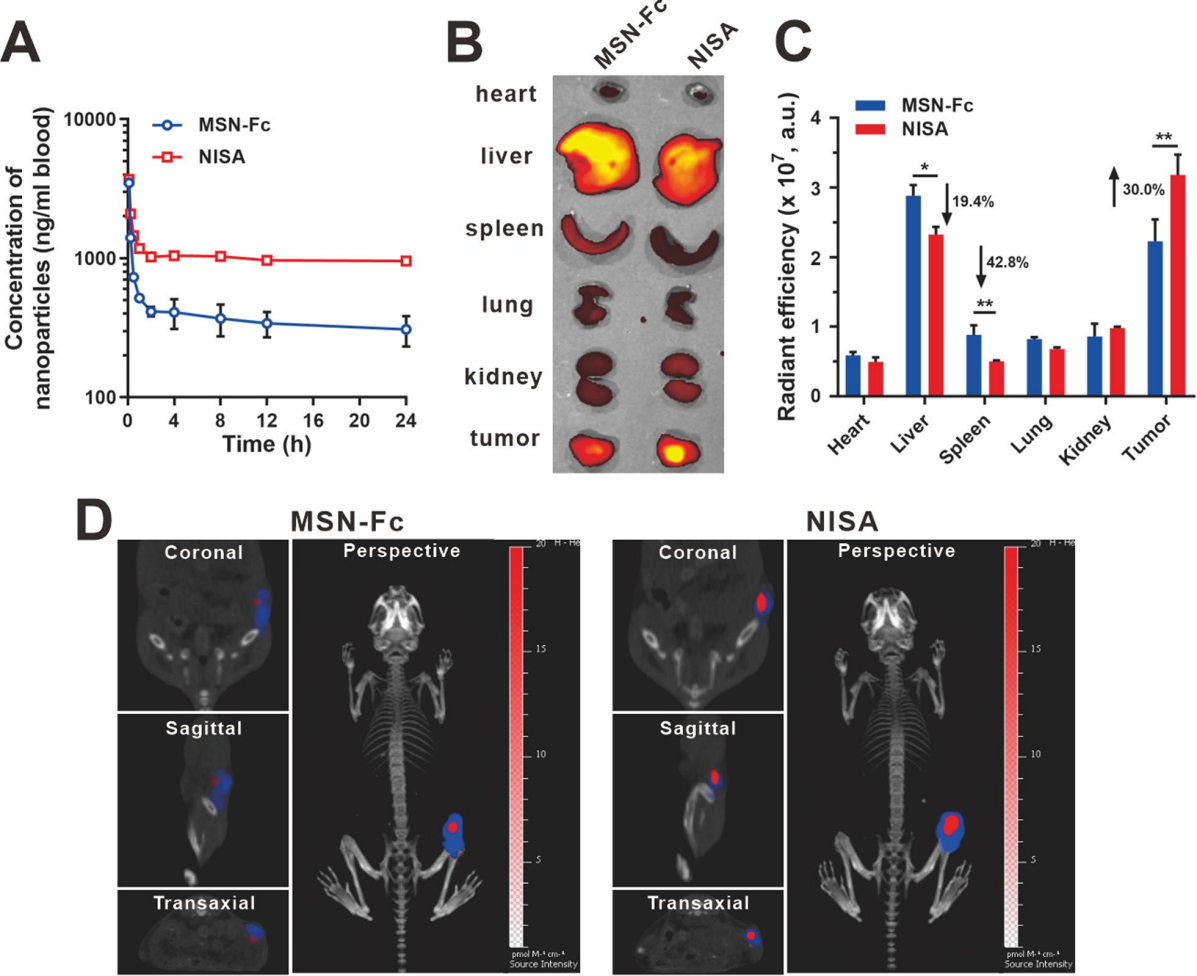
Blood clearance and in vivo tumor targeting of NISA. **A** Blood clearance curves of MSN-Fc and NISA. iFluor 647 was used as fluorescent probe for nanoparticle quantification. **B** Biodistribution of the nanoparticles. Ex vivo fluorescent imaging of the major organs were shown. (**C**) Statistical assay of the fluorescent intensity in **B**. **D** IVIS Spectrum CT multimodal imaging system was used to identify the tumor targeting of the nanoparticles. Blue and red signals indicated the tumor and nanoparticle, respectively. Images in the tumor regions from diverse angles (coronal, sagittal, transaxial, and perspective) were shown. Data are presented as mean ± SD (n = 3). *p < 0.05, **p < 0.01

Extended blood circulation is important for efficient in vivo tumor targeting. The blood clearances of iFluor 647-labeled MSN-Fc and NISA were evaluated in female BALB/c mice (Fig. 4A). Compared to MSN-Fc (half-life 15.0 ± 1.8 h), the inclusion of long-chain PEG_5000_ for Fc shielding in NISA (half-life 22.4 ± 1.6 h) contributed a more favorable circulation profile, which would benefit the tumor targeting in vivo. In contrast, free Fc was rapidly cleared from the circulation with a much shorter half-life (5.1 ± 0.5 h) (Additional file 1: Fig. S11).

As expected, NISA accumulated more in 4T1 tumors compared to MSN-Fc, and this effect is accompanied by 19.4% and 42.8% decreased distribution in liver and spleen, respectively (Fig. 4B, C). The proportion of the nanoparticle (iFluor 647-labeled MSN-Fc or NISA) dose distributed at the tumor site to the total injected dose was also examined. 6 h after the i.v. injection, the nanoparticle content in the tumor site was determined using an established standard curve between iFluor 647 fluorescence intensity and corresponding nanoparticle content according to the literature [44]. It showed that 1.2% of the injected MSN-Fc was obtained at the tumor site. In contrast, dramatically increased proportion of the injected NISA (2.2%) was found in the tumor, indicating the contribution of long-chain PEG_5000_ in Fc protection and tumor targeting (Additional file 1: Fig. S12). The tumor accumulation of the nanoparticles was also confirmed using a multimodal luminescence and X-ray computed tomography imaging system (Fig. 4D). 12 h after the iFluor 647-labeled nanoparticle injection, relatively lower fluorescent nanoparticle signal (red) was observed in the bioluminescent tumor sites (blue) in the MSN-Fc group. In contrast, increased colocalization of nanoparticles and tumors was obtained in NISA group, which was confirmed from diverse angles (coronal, sagittal, transaxial and perspective), indicating the improved tumor targeting conferred by the prolonged circulation of NISA.

### Anticancer effects of NISA in vivo

**Fig. 5:**
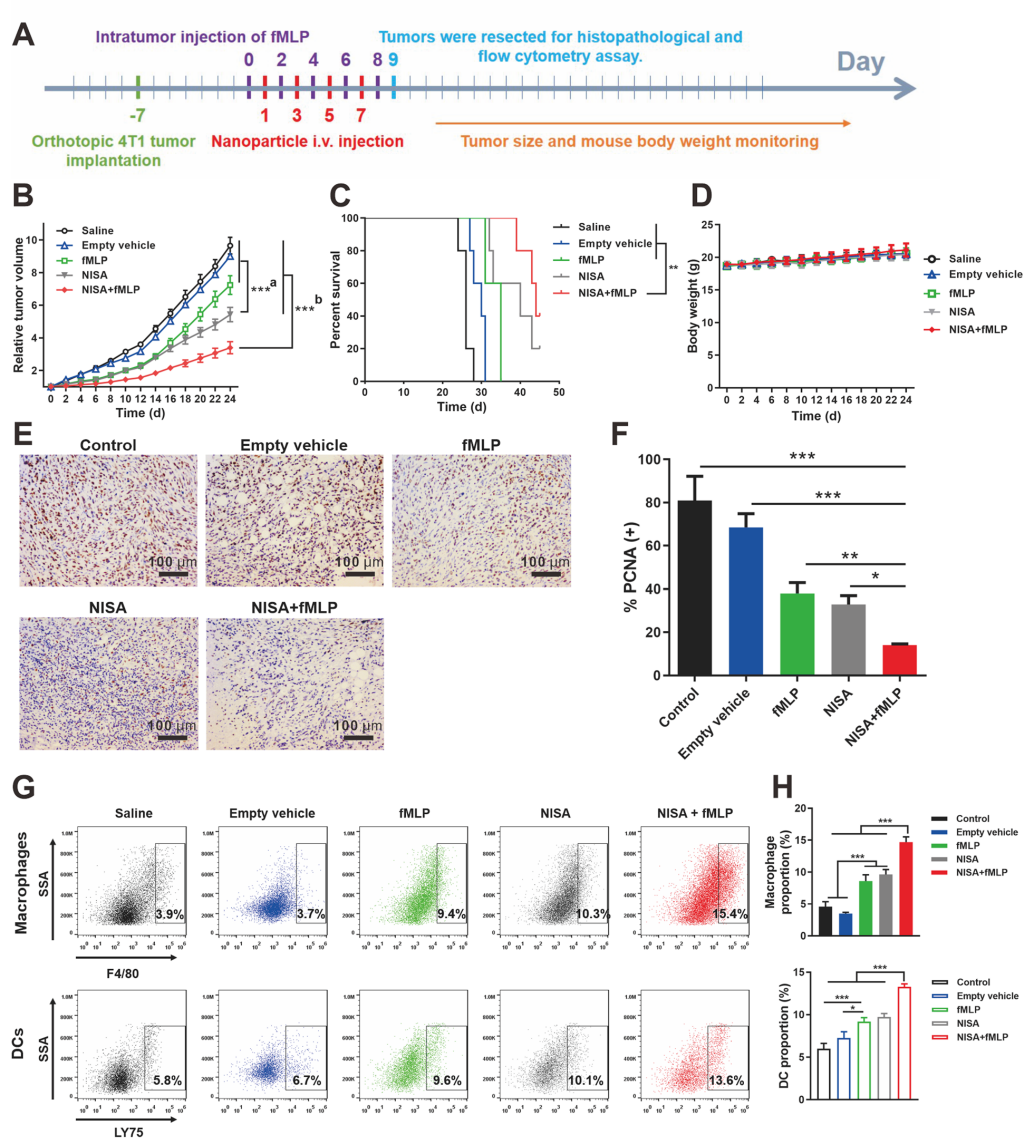
The antitumor effect of NISA in orthotopic 4T1-bearing mice. **A** Treatment regimen. **B** Tumor growth curve. a, NISA versus Saline, Empty vehicle, and fMLP. b, NISA + fMLP versus all other groups. **C** Mouse survival curve. **D** Mouse body weight. **E** Proliferative tumor cells (brown color) were stained with PCNA antibody. **F** Statistical assay of the PCNA positive tumor cells. **G** Representative flow cytometry profiles of macrophages and DCs in tumors after the indicated treatment. **H** Statistical assay of the immune cell proportions in **G**. Data are expressed as mean ± SEM in **B**, and mean ± SD in **C**, **D** and **F**. n = 5 for **B**–**D**. n = 3 for **F** and **H**. *p < 0.05, **p < 0.01, and ***p < 0.001

We then investigated the anticancer effects of NISA in vivo. The treatment procedure is shown in Fig. 5A. In the orthotopic 4T1 tumor-bearing mice, NISA significant delayed the tumor growth compared to empty vehicle and saline groups, which were actually ineffective. The combined usage of fMLP further enhanced the antitumor effect (Fig. 5B, Additional file 1: Fig. S13). fMLP can help recruit more immune effector cells to tumor sites, where they can be stimulated and activated by NISA, and led to enhanced antitumor efficacy. Compared with NISA, other controls, including free Fc, Fc + fMLP and MSN-Fc had poor antitumor effects (Additional file 1: Fig. S14), which may be ascribed to the rapid blood clearance of Fc and MSN-Fc from the circulation (Fig. 4A, Additional file 1: Fig. S11) and low distribution in the tumor site (Additional file 1: Fig. S12). The median survivals of the mice were extended from 26 d (Saline), to 35 d (fMLP), 40 d (NISA), and 44 d (NISA + fMLP), respectively (Fig. 5C). NISA + fMLP treatment earned the best efficacy in Increase in Life Span (ILS) of 69.2% (Additional file 1: Table S1). The mice body weight in all groups increased slightly in the therapeutic process, suggesting no overt toxicity (Fig. 5D). Histopathological examination showed that NISA led to less PCNA-positive cells, and this effect was more obvious when fMLP was simultaneously used (Fig. 5E, F). H&E staining of major organ including heart, liver, spleen, lung and kidney in all groups did not show obvious histological toxicity (Additional file 1: Fig. S15), suggesting that NISA were well-tolerated and biocompatible at the doses used, although further toxicity testing is warranted.

The flow cytometry analysis showed that NISA or fMLP treatment led to significantly increased number of macrophages and DCs in tumor sites compared to saline and empty vehicle (Fig. 5G, H**)**. We further identified macrophage polarization in the tumor site (Additional file 1: Fig. S16). It is known that M1-like phenotype cells are tumoricidal in contrast to the M2-like cells, which are tumor-supportive, and present as an effective target for immunotherapy. The proportion of M2-like cells in total macrophages was kept at a low level (below < 10%) in all groups. NISA did not obviously influence the proportion of M1-like cells, whereas the proportion of M1-like macrophages in NISA + fMLP group reached the highest level (~ 22%), which may confer a favorable antitumor effect. The activated CD11c^+^MHCII^+^ DCs in the tumor site were also significantly increased in NISA and NISA + fMLP groups (Additional file 1: Fig. S17), presenting an immune enhanced tumor microenvironment. Moreover, NISA or NISA + fMLP treatment increased the NK cells in tumor sites (Additional file 1: Fig. S18). Generally, NISA + fMLP resulted in the most efficient recruitment of the immune cells, which were consistent with its antitumor effect (Fig. 5).

## Conclusions

In conclusion, through conjugating multivalent Fc fragments on MSN surface, we developed a nanoparticle-based innate immune system activator, NISA. Distinct from previous efforts which mostly stimulated the adaptive immunity or only one components of innate immune system, NISA can activate multiple elements of innate system including the complement system and the diverse innate cells (macrophages and DCs). With 4T1 cells as the model, NISA exhibited excellent antitumor effects both in vitro and in vivo. The antitumor potency conferred by NISA highlighted the significance of simultaneously promoting multiple innate immune effectors in cancer therapy. As MSN already present a versatile platform for drug loading [45], NISA can be easily combined with other therapeutic modality (such as chemotherapy) for integrated multimodal synergistic therapy.

## Materials and methods

### Materials

Tetraethylorthosilicate (TEOS, 98%), cetrimonium bromide (CTAB, 98%), and matrix metalloproteinase-2 from mouse were purchased from Sigma-Aldrich (St. Louis, MO). Ethyl acetate was purchased from Sinopharm Chemical Reagent Company (Shanghai, China). *N*-(3-Dimethylaminopropyl)-3-ethylcarbodiimide hydrochloride (EDC) and *N*-hydroxysuccinimide (NHS) were obtained from Aladdin (Shanghai, China). Mouse IgG3 Fc fragments (~ 26 kDa) were purchased from Sino Biological (Beijing, China). MMP-2 cleavable peptide (GPLGIAGQC) was synthesized by GL Biochem (Shanghai, China). SH-PEG_2000_-COOH was purchased from JenKem Technology (Beijing, China). Methoxy-PEG_5000_-NHS were purchased from Ponsure Biotech (Shanghai, China). Guinea pig serum was purchased from Nanjing SenBeiJia Biological Technology (Nanjing, China). Dulbecco’s modified Eagle’s medium (DMEM), fetal bovine serum (FBS), PBS solution, penicillin and streptomycin were gained from Thermo Fisher Scientific (Waltham, MA). Double distilled water was purified using a Millipore simplicity system (Millipore, Bedford, MA). All other chemicals were of analytical grade and used without further purification.

4T1 mouse breast cancer cell line was obtained from Shanghai Model Organisms Center (Shanghai, China). Mouse macrophage RAW264.7 and dendritic cell DC2.4 [46] were kindly provided by Shanghai Institute of Immunology (Shanghai, China). 4T1 and RAW264.7 cells were cultured in DMEM medium with 10% FBS, 10^5^ U/L penicillin and 100 mg/L streptomycin. DC2.4 cells were cultured in 1640 medium with 10% FBS, 10^5^ U/L penicillin and 100 mg/L streptomycin. The culture was maintained at 37 °C in a humidified atmosphere containing 5% CO_2_.

Female BALB/c mice (~ 20 g) were provided by Shanghai Laboratory Animal Center (Chinese Academy of Sciences, Shanghai, China). The animal experiment designed in this study was approved by the IACUC of Shanghai Jiao Tong University School of Medicine (SJTU-SM).

### Preparation and characterization of NISA

The MSN was synthesized according to our previous report [44]. Briefly, 250 mL of CTAB solution (2 g/L) was added to a 500 mL round-bottom flask, and then 1.75 mL of NaOH solution (2 M) was added as catalyst. After the solution was heated to 70 ℃, 2.5 mL TEOS was added drop by drop and the solution was vigorously stirred. When the solution turned white, 2.5 mL ethyl acetate was added to terminate the reaction. After resting for 2 h, the solution was centrifuged (11,000 rpm 10 min) to collect MSN, which was washed with methanol for 3 times.

To sulfhydryl the MSN surface, 500 mg of the product was dissolved in 100 mL methanol. Then, 0.5 mL MPTMS was added and the solution was stirred in argon at 80 ℃ for 24 h. MSN-SH was obtained after removing the pore-generating template (CTAB) using 1% NaCl solution in methanol [47].

To synthesize the pyridyldithiol-terminated nanoparticles (MSN-s-s-PD), 25 mL methanol containing 250 mg MSN-SH was dropwise added into a 10 mL methanol solution containing 0.55 g 2,2′-dipyridyldisulfide and 0.2 mL glacial acetic acid. The mixed solution was stirred for 24 h at room temperature. The resulting MSN-s-s-PD was obtained by centrifugation (11,000 rpm, 10 min).

To conjugate Fc fragment to the nanoparticle surface, SH-PEG_2000_-COOH (0.6 mg) was added to MSN-s-s-PD (3 mg) in 10 mM HEPES buffer (pH 8.5), and the mixture was stirred at room temperature for 3 h. The resulting partially carboxyl-terminated particles (MSN-COOH) was obtained by centrifugation (11,000 rpm, 10 min). Then 3 mg MSN-COOH was re-dispersed in 500 µL PBS buffer (pH 8.6) containing EDC (0.3 mg/mL) and NHS (0.4 mg/mL). 50 µg mouse IgG3 Fc fragments were added to the solution. The mixture was stirred in the dark at room temperature for 6 h. The resulting Fc-conjugated nanoparticles (MSN-Fc) was obtained by centrifugation (11,000 rpm, 10 min). The loading efficiency of IgG3 Fc was calculated through quantifying the Fc proteins on the nanoparticles using Bradford protein assay compared to the feeding amount.

To shield the conjugated Fc fragment, we modified the nanoparticles with the MMP-2 cleavable long-chain PEG_5000_. Briefly, 1.56 mg of MMP-2 cleavable peptide (GPLGIAGQC) [32] and 1.92 mg of Methoxy-PEG_5000_-NHS were dissolved in PBS buffer (pH 8.6, containing EDC 0.3 mg/mL, NHS 0.4 mg/mL). The mixture was stirred in the dark at room temperature for 12 h. The resulting Methoxy-PEG_5000_-GPLGIAGQC was purified through ultrafiltration using a MW cut-off of 3000 Da membrane to remove the free peptides, and then dropwise added to the HEPES solution containing 3 mg of MSN-Fc. The mixture was stirred in the dark at room temperature for 24 h. The resulting long-chain PEG_5000_ shielded nanoparticles loading Fc (NISA) were obtained by centrifugation (11,000 rpm, 10 min). For nanoparticle fluorescence labeling, iFluor 647-labeled MSN was prepared as we previously described [48].

Transmission electron microscopy (TEM) micrographs were performed on an FEI Talos F200X system. The hydrodynamic size and zeta potential of nanoparticles were determined through dynamic light scattering (DLS) method and measured by ZetaSizer Nano ZS instrument (Malvern, Worcestershire, UK). X-ray photoelectron spectroscopy (XPS) was used to confirm the sequential modification and loading of the functional molecules and IgG3 Fc [37]. To examine the stability of Fc on the nanoparticles, 2 mg NISA containing iFluor 647-labled Fc was dispersed in 6 ml PBS with 10% FBS, and then shaken at 250 rpm and 37 ℃. After 1, 2, 4, 8, 12 and 24 h, the mixture was centrifuged (11,000 rpm, 30 min), and the fluorescence of the supernatant was detected to identify Fc shedding and estimate its retention.

### Cleavage of MMP-2 sensitive peptide

The functions of MMP2-cleavable peptide were evaluated by enzymatic digestion using the active MMP-2 [32]. To detect the digested long-chain PEG_5000_, FITC-PEG_5000_-NHS was used instead of methoxy-PEG-NHS for the nanoparticle preparation. The FITC-labeled NISA (1 mg/mL) were incubated with MMP-2 (5 µg/mL, i.e. 0.07 µM) in pH 7.4 HEPES-buffered saline containing 10 mM CaCl_2_ at 37 ℃ for 12 h. The digested fragments (FITC-PEG_5000_) were identified by measuring the concentration of FITC by microplate reader (Ex 488 nm, Em 520 nm).

### Activation of innate immune cells (RAW264.7 and DC2.4)

To identify the targeted binding of the nanoparticles to the innate immune cells, RAW264.7 and DC2.4 cells were seeded into 24-well plates at a density of 150,000 cells per well, respectively. After 12 h culture, the cell medium was replaced with fresh medium containing iFluor 647-labeled MSN-Fc (Fc 50 µg/mL) for 1 h incubation. Then, the cells were examined by confocal microscopy (Ex 633 nm, Em 650 nm) and flow cytometry (Ex 637 nm, channel RL1 670/14 nm, Attune NxT Flow Cytometer, Thermo Fisher Scientific). For the blocking test, free Fc (50 µg) were added together with MSN-Fc nanoparticles.

The ERK pathway activation by MSN-Fc was examined using western blot assay. RAW264.7 and DC2.4 cells were seeded into 6-well plates at a density of 500,000 cells per well, respectively. After 12 h culture, the cells were treated with MSN-Fc (Fc 50 µg/mL) for 2 h incubation. Then, the cell proteins were extracted, quantified, and processed for western blot assay. p44/42 MAPK (Erk1/2) rabbit mAb and phospho-p44/42 MAPK (Erk1/2) rabbit mAb (Cell Signaling Technology) were used for Erk detection. The downstream NF-κB was also examined. The cells were incubated with MSN-Fc (Fc 50 µg/mL) for 2 h. After 2 h, the cells were fixed with 4% paraformaldehyde, and incubated with NF-κB p65 rabbit mAb (Cell Signaling Technology) at 4 ℃ for 24 h. Then, the cells were incubated with Alexa Fluor 488 (for RAW264.7 assay) or iFluor 647 (for DC2.4 assay)-labeled second antibody (Cell Signaling Technology) for 1 h, and then observed under microscope. MSN-COOH or free Fc was included as the control.

For the assay of cytokine production, RAW264.7 and DC2.4 cells were cultured in 24-well plates with the medium containing the nanoparticles (MSN-Fc or MSN-COOH, 200 µg/mL). After 24 h, the TNF-*α* and IL-6 levels in the supernatant were quantified according to the manufacturer’s instructions using mouse TNF-*α* and IL-6 Elisa Kit (MultiSciences, Hangzhou, China).

### Activation of complement system and cytotoxicity to 4T1 cells

MSN-COOH, MSN-Fc and NISA (200 µg/mL) were incubated with guinea pig serum at 37 ℃ for 30 min. Then, the nanoparticles were centrifuged and the C3a levels in the supernatant serum were quantified using the Guinea pig C3a Elisa Kit (Fankel Bio, Shanghai, China) [49]. NISA pre-incubated with MMP-2 (5 µg/mL) for 12 h at 37 ℃, indicated as NISA + fMLP, and 4T1 cells (10^5^ cells in 24 well plates) were also included as controls.

For the cytotoxicity assay, 4T1 cells (5000 per well) suspended in 250 µL mixed medium (100 µL guinea pig serum plus 150 µL DMEM) containing various concentrations of MSN-Fc or MSN-COOH (20, 50, 100, 200, 400 µg/mL) were added to 96-well plates. After 24 h incubation, the floating dead cells in the medium supernatant were counted. The activity of lactic dehydrogenase (LDH) in the medium was detected according to the manufacturer’s instructions using LDH assay kit (Beyotime Biotechnology, Shanghai, China). The viability of the adherent cells on the plate bottom was measured using Cell Counting Kit-8 (Dojindo Laboratories, Kumamoto, Japan).

### Blood clearance kinetics

iFluor 647-labeled nanoparticles (NISA or MSN-Fc, 50 mg/kg) were injected to the mice through caudal vein. At the designated time points, 100 µL blood was taken from the orbital vein to quantify the fluorescence signal along with the time. The standard curve between iFluor 647 fluorescence intensity and corresponding nanoparticle content was used to determine the content of nanoparticles in blood as we previously described [44].

### Orthotopic tumor targeting and biodistribution

4T1 tumor-bearing mice were injected through the caudal vein with iFluor 647-labled NISA or MSN-Fc (30 mg/kg) at iFluor 647 dose of ~ 0.25 mg/kg, respectively. After 12 h, the mice were intraperitoneally injected with d-luciferin (100 mg/kg, J&K Chemical, China). 10 min later, the mice were anesthetized and imaged under the IVIS Spectrum/CT imaging system (PerkinElmer, USA) to monitor bioluminescence and fluorescence signal (Ex 605 nm, Em 680 nm). Then, the mice were sacrificed. The tumor and major organs (heart, liver, spleen, lung, and kidney) were excised for ex vivo imaging.

We further examined the proportion of the nanoparticle dose at the tumor site to the injected total dose. 600 µg nanoparticles (MSN-Fc or NISA) labeled with iFluor 647 were i.v. injected to 4T1 tumor-bearing mice. After 6 h, the tumors were excised and homogenized. The nanoparticle dose in the tumor was determined using the established standard curve between the iFluor 647 fluorescence intensity (Ex 605 nm, Em 660 nm) and the corresponding nanoparticle content according to the method described [44].

### Mouse model and treatment protocol

Female BALB/c mice were inoculated with 4T1 cells (1 × 10^6^) into the right fourth mammary fat pad. When the tumors reached ~100 mm^3^ (day 0), mice were randomly divided into five groups (n= 5) and treated with (1) saline (control), (2) Empty vehicle (nanoparticles containing all components of NISA except Fc), (3) fMLP, (4) NISA, and (5) NISA + fMLP. fMLP (100 nM in 50 µL PBS) was intratumorally injected on day 0, 2, 4, 6, 8. The nanoparticles (0.75 mg) loading Fc (12.5 µg) was intravenously administered to the mice through the tail vein on day 1, 3, 5, and 7, respectively. The tumor volume and mice body weight were monitored throughout the study. Tumor volumes (mm^3^) were calculated as 1/2 × length × width^2^. Survival was recorded until tumor volume reached ethical limit (2000 mm^3^) [50]. Increase in life span (*ILS*) was obtained using the formula: % *ILS* = (T/C − 1) × 100%. T and C are median survival time of the mice in the treated and control group, respectively.

In a separate study, 24 h after the final injection (day 9), 3 mice from each group were sacrificed. The tumors were removed and processed for paraffin sections for immunohistochemical assay. Other major organs included heart, liver, spleen, lung and kidney were collected for H&E histological assay for toxicity evaluation.

### Flow cytometry analysis

In another study, 3–4 mice from each group at day 9 were sacrificed and the tumors were harvested and digested with collagenase I and DNase to generate single-cell suspensions. Then, the cells were collected and diluted to 1 × 10^7^ cells/mL. 100 µL cells were stained using fluorescent conjugated antibodies. Macrophages were labeled with rat anti-mouse F4/80 antibody (PE) (BD Biosciences, San Diego, CA) and also hamster anti-mouse CD11c (PerCP-Cy 5.5) and rat anti-mouse CD206 antibody (Alexa Fluor 647) (BD Biosciences, San Diego, CA) to identify the polarization [51]. DCs were marked with rat anti-mouse LY75/DEC-205 antibody (FITC) (Abcam, Hong Kong) [52]. The activated DCs were further identified with hamster anti-mouse CD11c (PerCP-Cy5.5) and rat anti-mouse MHCII (Alexa Fluor 488) antibodies (BD Biosciences, San Diego, CA) [53, 54]. The flow cytometry assay was performed using Attune NxT Flow Cytometer (Thermo Fisher Scientific). Data wer analyzed using FlowJo software (FlowJo, Ashland, OR).

### Statistical analysis

Statistical analysis was conducted using GraphPad Prism 5.0 software (La Jolla, CA). Differences between groups were examined using Student’s *t*-test or ANOVA with Tukey’s multiple comparison tests. Differences were considered significant if *p*-value was less than 0.05.

## Supplementary Information

**Additional file 1: Figure S1. **TEM image of nude MSN without PEGylation and Fcfragments. **Figure S2. **Representative size distribution profiles of MSN-SH,MSN-COOH, MSN-Fc, and NISA. **Figure S3. **Surfaceelement components of the nanoparticles were detected using X-ray photoelectronspectroscopy (XPS) assay. The changes of surface N1s confirmed the sequentialmodification and loading of the functional molecules and IgG3 Fc. **FigureS4. **Fc conjugation efficiency along with increased feeding amount. Data areexpressed as means ± SD (n = 3). **Figure S5. **Examination of the left IgG3 Fc in the supernatantof MSN-Fc using SDS-PAGE. **Figure S6.** IgG3 Fc retention on NISA in PBScontaining 10% FBS at 37 ℃. Data are expressed as means ± SD (n = 3). **FigureS7. **Colloid stability of NISA in PBS at 4 ℃ for 7 days. Data are expressedas means ± SD (n = 3). **Figure S8.** Fcγ receptors on the cell membrane ofmacrophages (RAW264.7) and dendritic cells (DC2.4). Fcγ receptors wasidentified using anti-CD64/FcγR rabbit polyclonal antibody (1:500, SinoBiological, Beijing, China) and Alexa Fluor 488-labeled goat anti-rabbit IgG secondaryantibody (1:1000, Abcam, Shanghai, China) for RAW264.7 or Alexa Fluor 647-labeledgoat anti-rabbit IgG secondary antibody (1:1000, ThermoFisher, Shanghai, China)for DC2.4. **Figure S9. **Examination of ERK activation in the cells treatedwith free Fc or MSN-Fc. (**A**) Western blot assay of p-ERK expression inRAW264.7 and DC2.4 cells. Statistical assay (**B**, **C**) of p-ERK expressionin panel A. Data are expressed as means ± SD (n = 3). **Figure S10.**MSN-Fc treatment induced the formation of more pseudopodiums in RAW264.7 andDC2.4 cells. The cell pseudopodiums were observed through the immunofluorescentstaining of F-actin using acti-stain 670 phalloidin. Notedthat TNF-*α* (green color) in the cells was stained with rabbitanti-TNF-*α *antibody (Abcam, Hong Kong),and Alexa Fluor 546 donkey anti-rabbit IgG (Thermo Fisher, Waltham)and observed at Ex 556 nm and Em 573 nm. **FigureS11. **Blood clearance curve of free Fc (iFluor 647 labeled). Thestandard curve between iFluor 647 fluorescence intensity and corresponding Fcconcentration was used to determine the Fc content in blood. Data are expressedas means ± SD (n = 5). **Figure S12. **Quantification of nanoparticle content in tumors.The dose of MSN-Fc (**A**) and NISA (**B**) in the tumors was determinedusing the established standard curve between the iFluor 647 fluorescenceintensity and the corresponding nanoparticle (MSN-Fc or NISA) concentrations.#1, #2, and #3 were three separate tumor samples. 6 h after injection, 1.2% ofthe injected MSN-Fc was obtained at the tumor site. In contrast, dramaticallyincreased proportion of the injected NISA (2.2%) was found in the tumor. **FigureS13.** Individual tumor growth curves of the mice in each group. **Figure S14.**The antitumor effect of Fc, Fc + fMLP and MSN-Fc in orthotopic 4T1-bearingmice. (**A**) Tumor growth curve (n = 5, mean ± SEM).S. a, NISA versus MSN-Fc. b, NISA versus Saline, Fc, and Fc + fMLP. c, NISA + fMLPversus all other groups. (**B**) Mouse body weight (n = 5, mean ± SD). Thegroups of saline, NISA, and NISA + fMLP indicated with dotted blue lines werealso displayed in Fig. 5 in the main text. **Figure S15.** On day 9 (24 hafter the final injection), 3 mice from each group were sacrificed, and themajor organs (heart, liver, spleen, lung, and kidney) of the mice were removedand processed for paraffin sections and histopathological examination (H&Estaining). **Figure S16. **Macrophagepolarization analysis. (**A**) Macrophages are defined as F4/80^+^cell. M1-like cells are F4/80^+^CD11c^+^CD206^−^,whereas M2-like cells are F4/80^+^CD11c^−^CD206^+^cells. (**B**) Statistical assay of the M1- and M2-like cell proportion intotal macrophages. Data are expressed as mean ± SD. n = 3. ***p < 0.001. **Figure S17. **Analysisof mature DCs in the tumors after various treatments. (**A**) Mature DCswere identified as LY75^+^CD11c^+^MHCII^+^.(**B**) Statistical assay of the mature DC proportions. Data areexpressed as mean ± SD (n = 4) ***p < 0.001. **FigureS18.** Examination of NK cells in tumors after theindicated treatments. (**A**) Representative flow cytometry profiles of NK cells in tumorsafter the indicated treatment. NK cells were marked with goat anti-mouseNKp46/NCR1 antibody (FITC) (R&D, Minneapolis, MN). (**B**) Statisticalassay of the NK cell percentage. Data are expressed as mean ± SD. n = 3. *p < 0.05, **p < 0.01. **Table S1. **Survival analysis of themice with various treatments.

## Data Availability

Most of the datasets supporting the conclusions of this article are included within this article and the additional files. The datasets used or analyzed during the current study are available on reasonable request.
