# Supplementary material for: A nano-innate immune system activator for cancer therapy in a 4T1 tumor-bearing mouse model
Source: J Nanobiotechnology. 2022 Jan 29;20:54. doi: 10.1186/s12951-022-01265-4 (PMC8800325; doi:10.1186/s12951-022-01265-4)
Supplement: Supplementary file 1 — Additional file 1: Figure S1. TEM image of nude MSN without PEGylation and Fcfragments. Figure S2. Representative size distribution profiles of MSN-SH,MSN-COOH, MSN-Fc, and NISA. Figure S3. Surfaceelement components of the nanoparticles were detected using X-ray photoelectronspectroscopy (XPS) assay. The changes of surface N1s confirmed the sequentialmodification and loading of the functional molecules and IgG3 Fc. FigureS4. Fc conjugation efficiency along with increased feeding amount. Data areexpressed as means ± SD (n = 3). Figure S5. Examination of the left IgG3 Fc in the supernatantof MSN-Fc using SDS-PAGE. Figure S6. IgG3 Fc retention on NISA in PBScontaining 10% FBS at 37 ℃. Data are expressed as means ± SD (n = 3). FigureS7. Colloid stability of NISA in PBS at 4 ℃ for 7 days. Data are expressedas means ± SD (n = 3). Figure S8. Fcγ receptors on the cell membrane ofmacrophages (RAW264.7) and dendritic cells (DC2.4). Fcγ receptors wasidentified using anti-CD64/FcγR rabbit polyclonal antibody (1:500, SinoBiological, Beijing, China) and Alexa Fluor 488-labeled goat anti-rabbit IgG secondaryantibody (1:1000, Abcam, Shanghai, China) for RAW264.7 or Alexa Fluor 647-labeledgoat anti-rabbit IgG secondary antibody (1:1000, ThermoFisher, Shanghai, China)for DC2.4. Figure S9. Examination of ERK activation in the cells treatedwith free Fc or MSN-Fc. (A) Western blot assay of p-ERK expression inRAW264.7 and DC2.4 cells. Statistical assay (B, C) of p-ERK expressionin panel A. Data are expressed as means ± SD (n = 3). Figure S10.MSN-Fc treatment induced the formation of more pseudopodiums in RAW264.7 andDC2.4 cells. The cell pseudopodiums were observed through the immunofluorescentstaining of F-actin using acti-stain 670 phalloidin. Notedthat TNF-α (green color) in the cells was stained with rabbitanti-TNF-α antibody (Abcam, Hong Kong),and Alexa Fluor 546 donkey anti-rabbit IgG (Thermo Fisher, Waltham)and observed at Ex 556 nm and Em 573 nm. FigureS11. Blood clearance curve [file 12951_2022_1265_MOESM1_ESM.docx]

**Supporting Information**

**A nano-innate immune system activator for cancer therapy in a 4T1 tumor-bearing mouse model**

Xiang-Yu Liu^1†^, Mao-Hua Zhu^1†^, Xiao-Yu Wang^1^, Xiao Dong^1^, Hai-Jun Liu^1^, Rui-Yang Li^1^, Shi-Chong Jia^1^, Qin Lu^1^, Mei Zhao^2^, Peng Sun^3^, Hong-Zhuan Chen^4^ and Chao Fang^1,5,*^

**
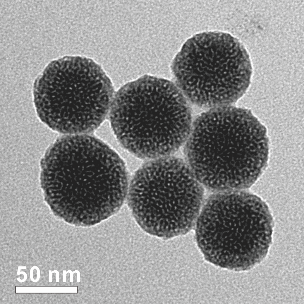
**

**Figure S1.** TEM image of nude MSN without PEGylation and Fc fragments.

**
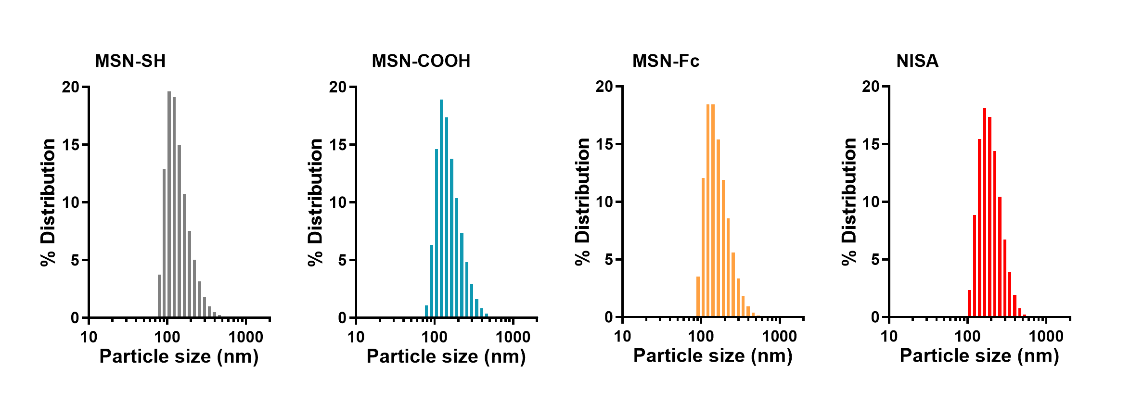
**

**Figure S2.** Representative size distribution profiles of MSN-SH, MSN-COOH, MSN-Fc, and NISA.


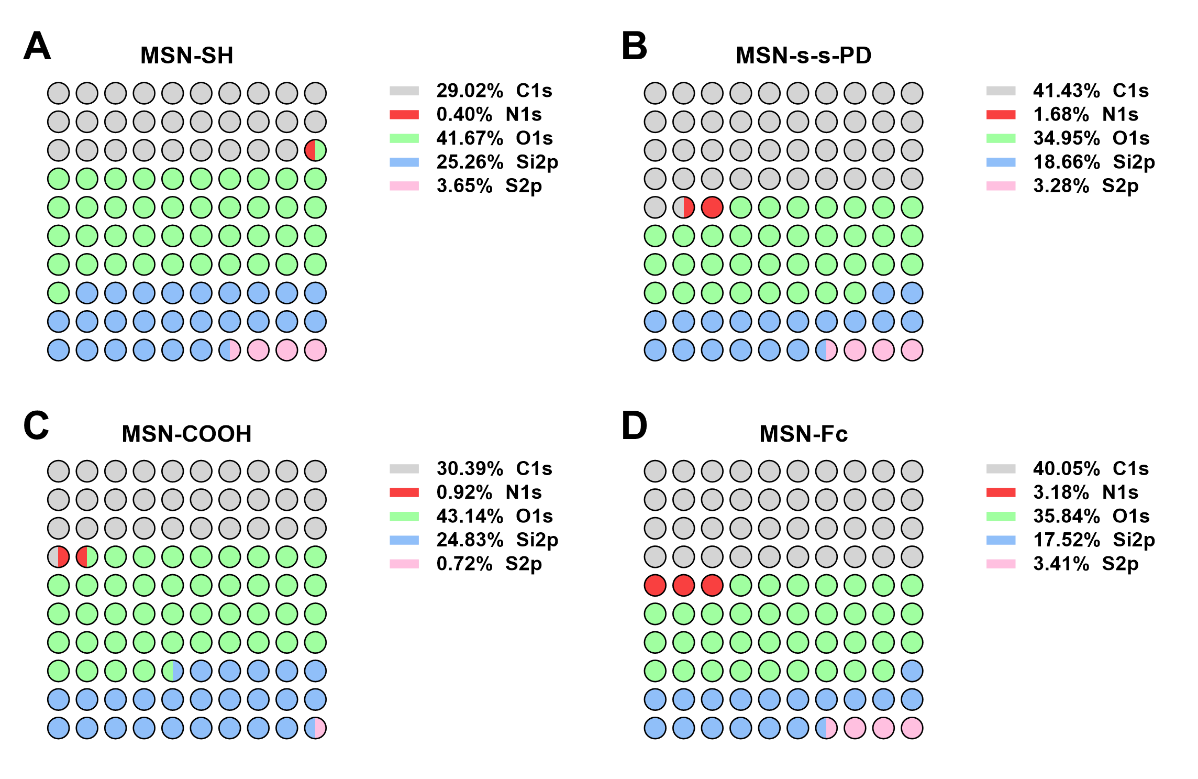


**Figure S3.** Surface element components of the nanoparticles were detected using X-ray photoelectron spectroscopy (XPS) assay. The changes of surface N1s confirmed the sequential modification and loading of the functional molecules and IgG3 Fc.

**
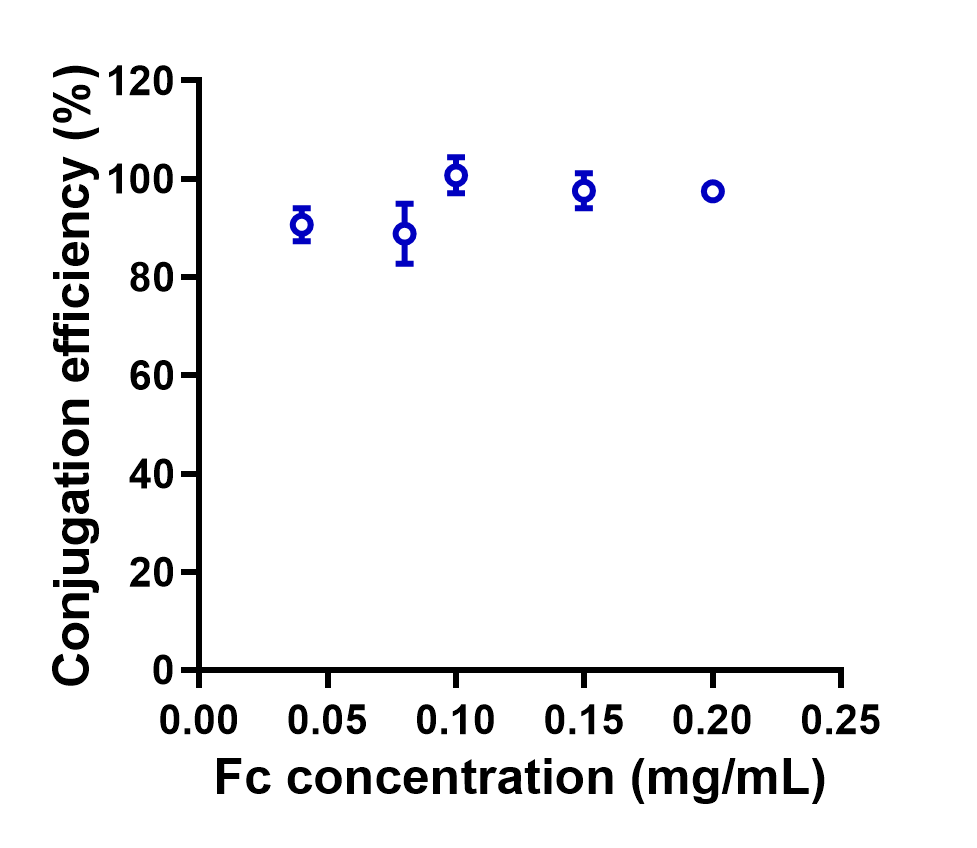
**

**Figure S4.** Fc conjugation efficiency along with increased feeding amount. Data are expressed as means±SD (n=3).


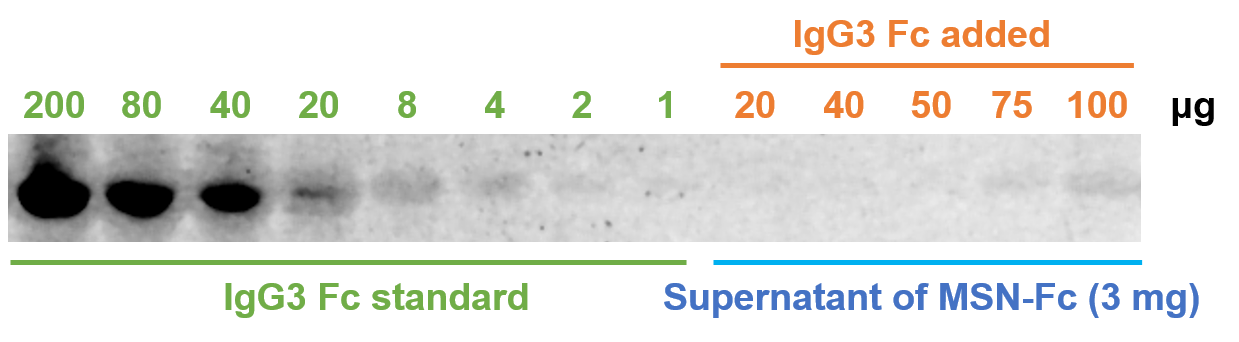


**Figure S5.** Examination of the left IgG3 Fc in the supernatant of MSN-Fc using SDS-PAGE.


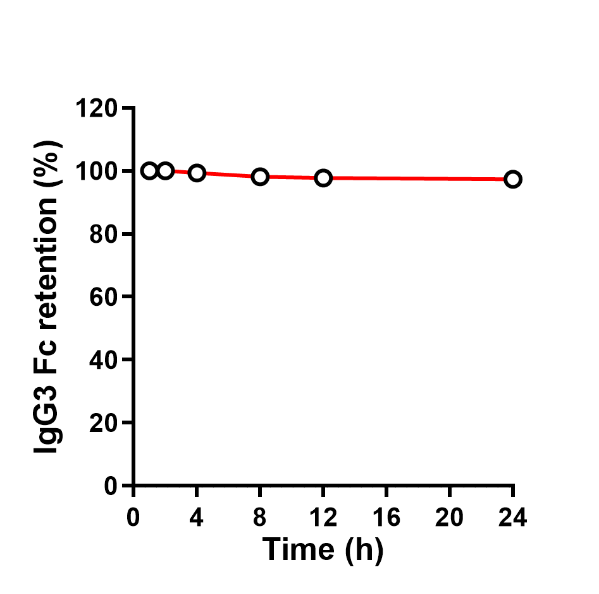


**Figure S6.** IgG3 Fc retention on NISA in PBS containing 10% FBS at 37 ℃. Data are expressed as means±SD (n=3).


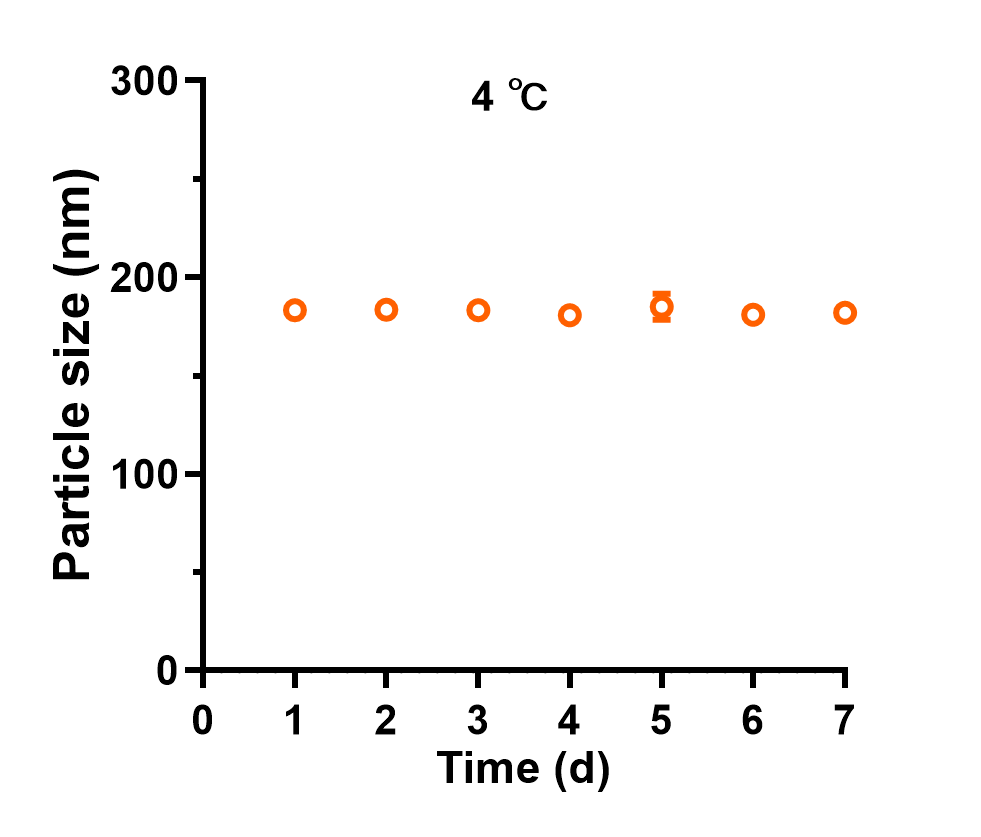


**Figure S7.** Colloid stability of NISA in PBS at 4 ℃ for 7 days. Data are expressed as means±SD (n=3).


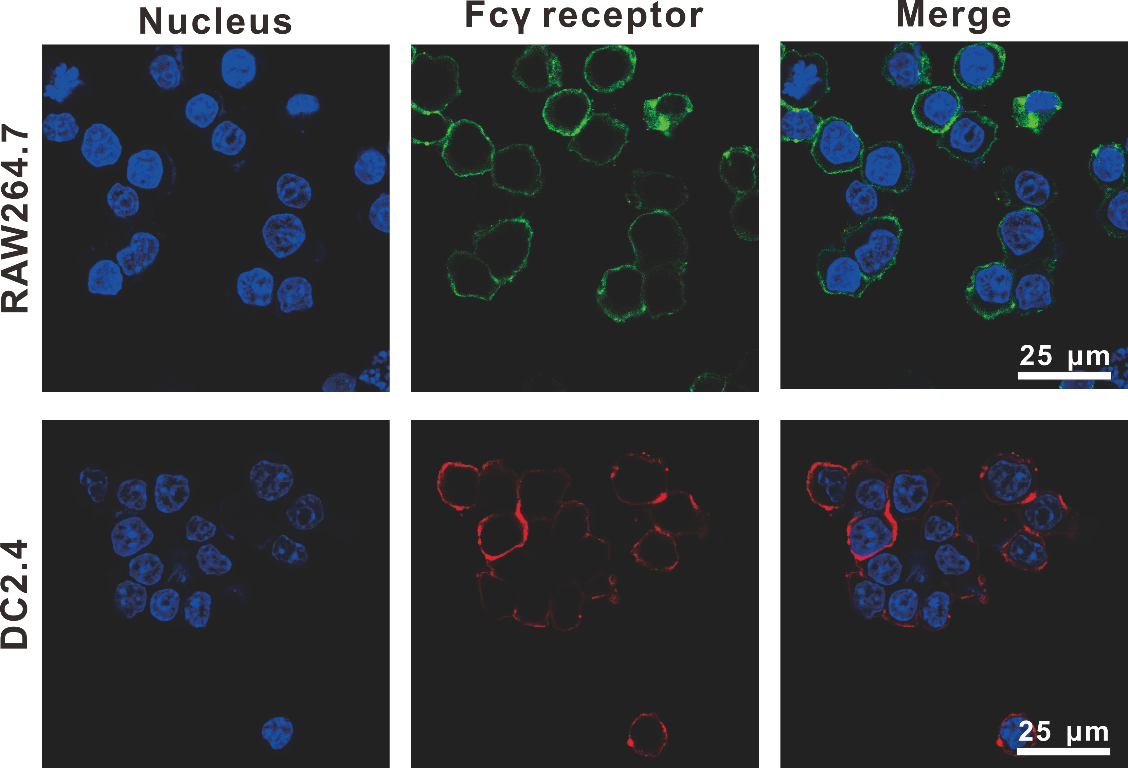


**Figure S8.** Fcγ receptors on the cell membrane of macrophages (RAW264.7) and dendritic cells (DC2.4). Fcγ receptors was identified using anti-CD64/FcγR rabbit polyclonal antibody (1:500, Sino Biological, Beijing, China) and Alexa Fluor 488-labeled goat anti-rabbit IgG secondary antibody (1:1000, Abcam, Shanghai, China) for RAW264.7 or Alexa Fluor 647-labeled goat anti-rabbit IgG secondary antibody (1:1000, ThermoFisher, Shanghai, China) for DC2.4.

**
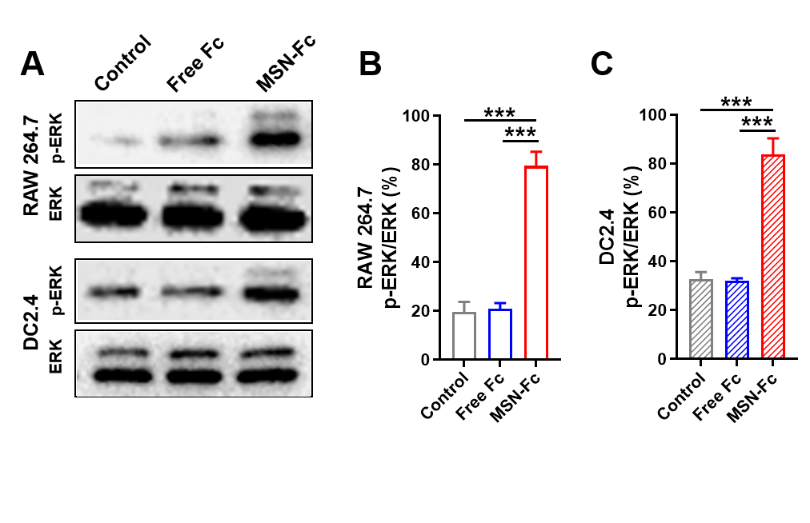
**

**Figure S9.** Examination of ERK activation in the cells treated with free Fc or MSN-Fc. (**A**) Western blot assay of p-ERK expression in RAW264.7 and DC2.4 cells. Statistical assay (**B, C**) of p-ERK expression in panel A. Data are expressed as means±SD (n=3).


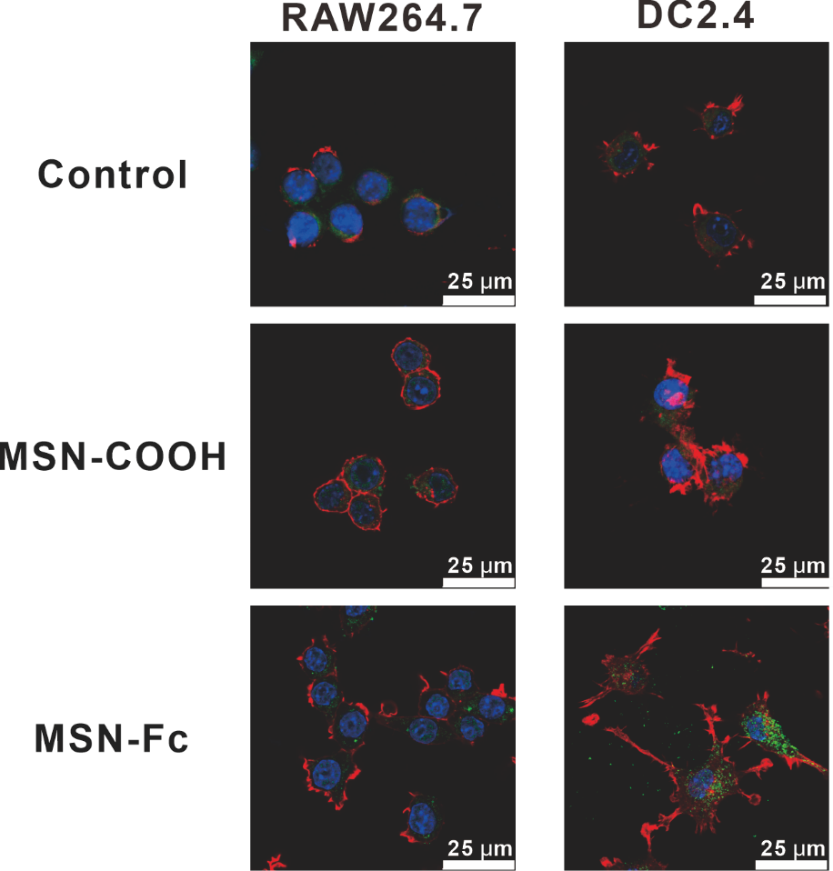


**Figure S10.** MSN-Fc treatment induced the formation of more pseudopodiums in RAW264.7 and DC2.4 cells. The cell pseudopodiums were observed through the immunofluorescent staining of F-actin using acti-stain 670 phalloidin. Noted that TNF-*α* (green color) in the cells was stained with rabbit anti-TNF-*α* antibody (Abcam, Hong Kong), and Alexa Fluor 546 donkey anti-rabbit IgG (Thermo Fisher, Waltham) and observed at Ex 556 nm and Em 573 nm.


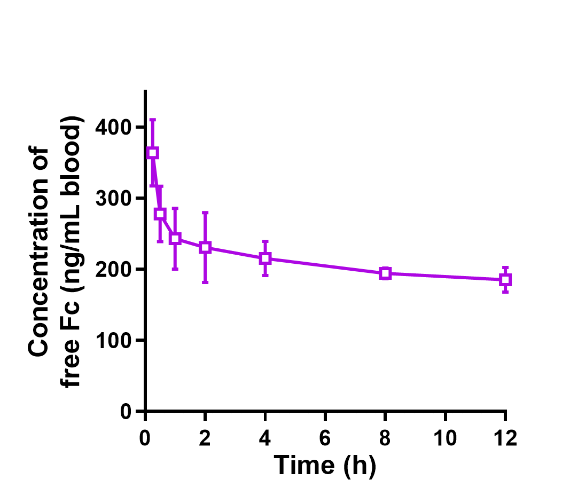


**Figure S11.** Blood clearance curve of free Fc (iFluor 647 labeled). The standard curve between iFluor 647 fluorescence intensity and corresponding Fc concentration was used to determine the Fc content in blood. Data are expressed as means±SD (n=5).


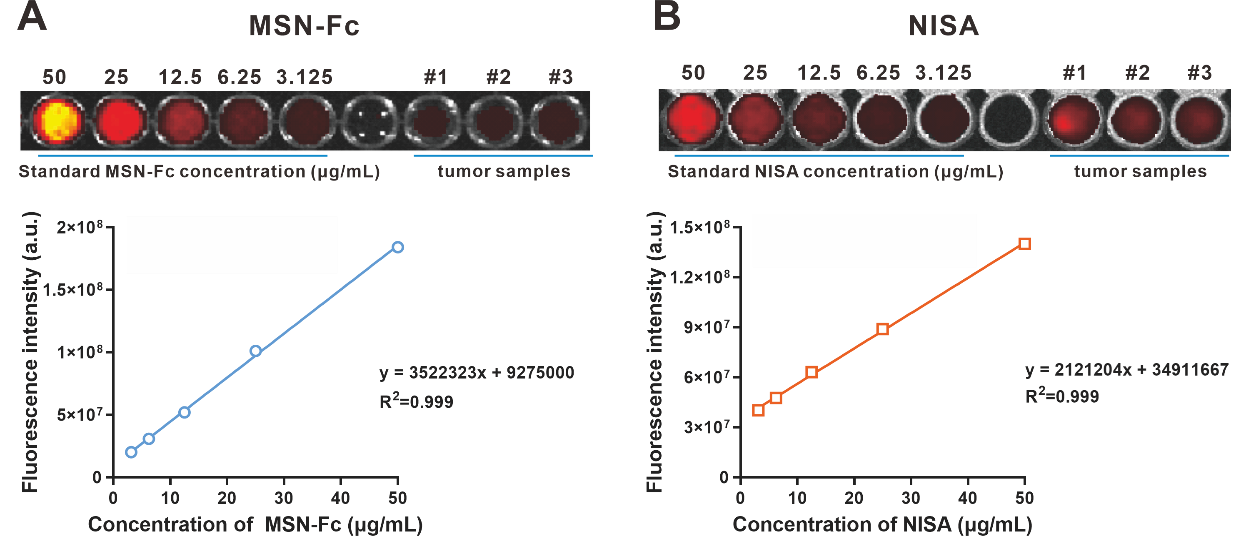


**Figure S12.** Quantification of nanoparticle content in tumors. The dose of MSN-Fc (**A**) and NISA (**B**) in the tumors was determined using the established standard curve between the iFluor 647 fluorescence intensity and the corresponding nanoparticle (MSN-Fc or NISA) concentrations. #1, #2, and #3 were three separate tumor samples. 6 h after injection, 1.2% of the injected MSN-Fc was obtained at the tumor site. In contrast, dramatically increased proportion of the injected NISA (2.2%) was found in the tumor.


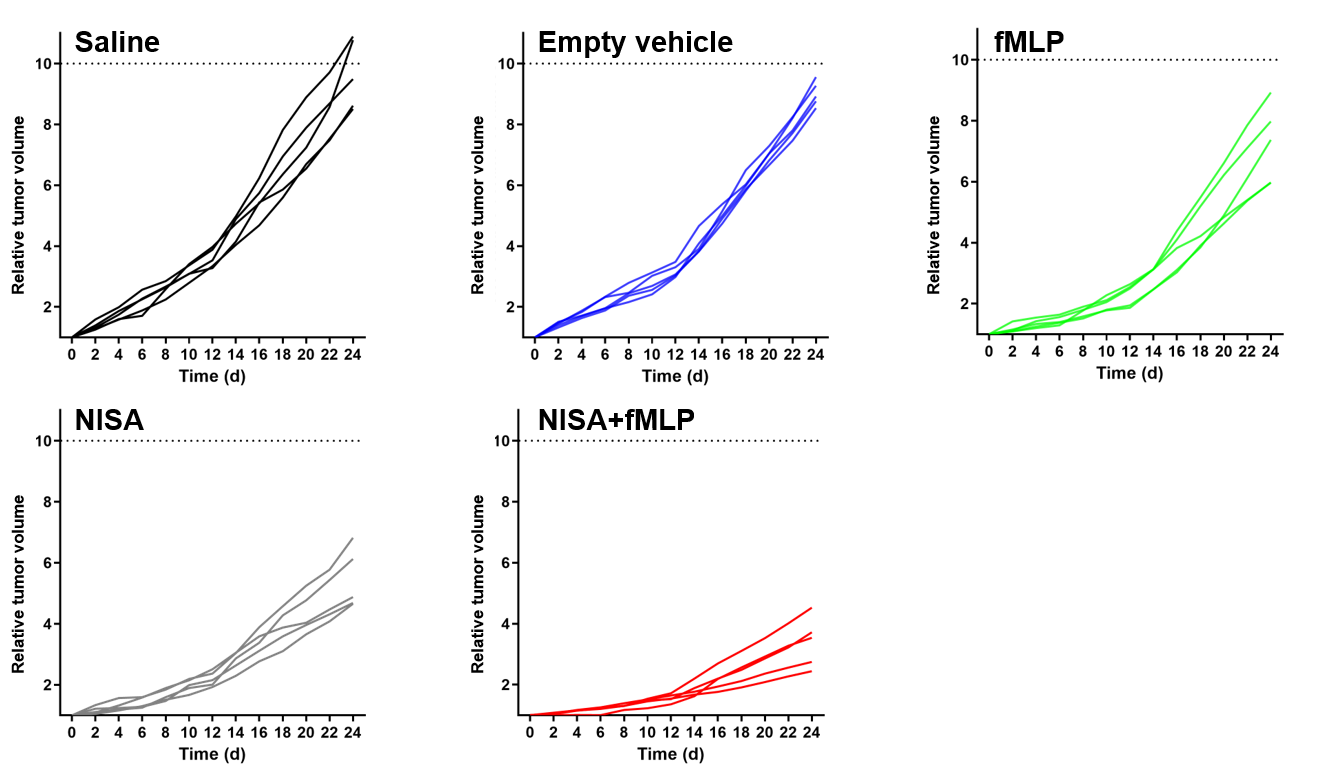


**Figure S13.** Individual tumor growth curves of the mice in each group.


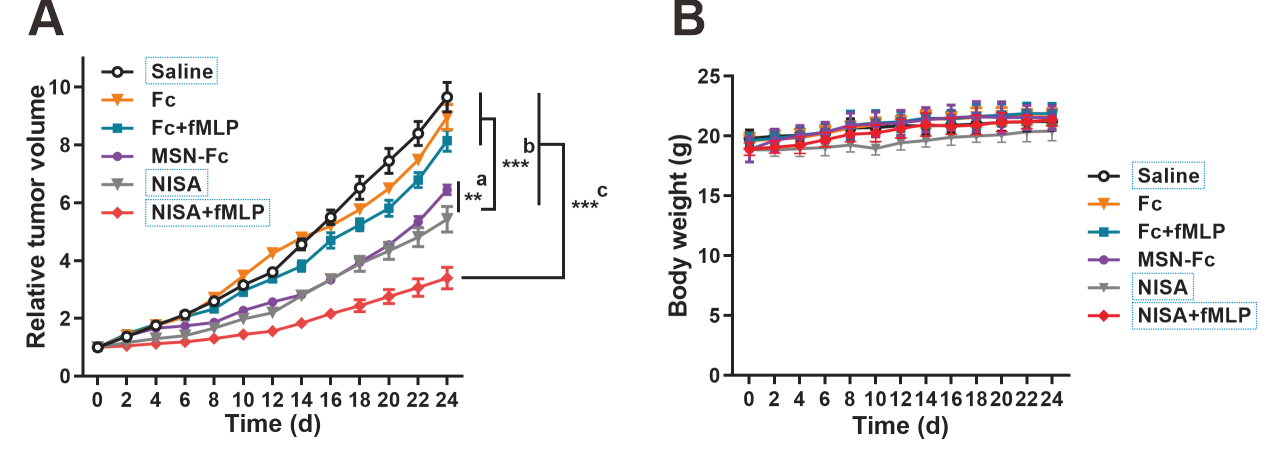


**Figure S14.** The antitumor effect of Fc, Fc+fMLP and MSN-Fc in orthotopic 4T1-bearing mice. (**A**) Tumor growth curve (n = 5, mean ± SEM). S. a, NISA versus MSN-Fc. b, NISA versus Saline, Fc, and Fc+fMLP. c, NISA+fMLP versus all other groups. (**B**) Mouse body weight (n = 5, mean ± SD). The groups of saline, NISA, and NISA+fMLP indicated with dotted blue lines were also displayed in Fig. 5 in the main text.


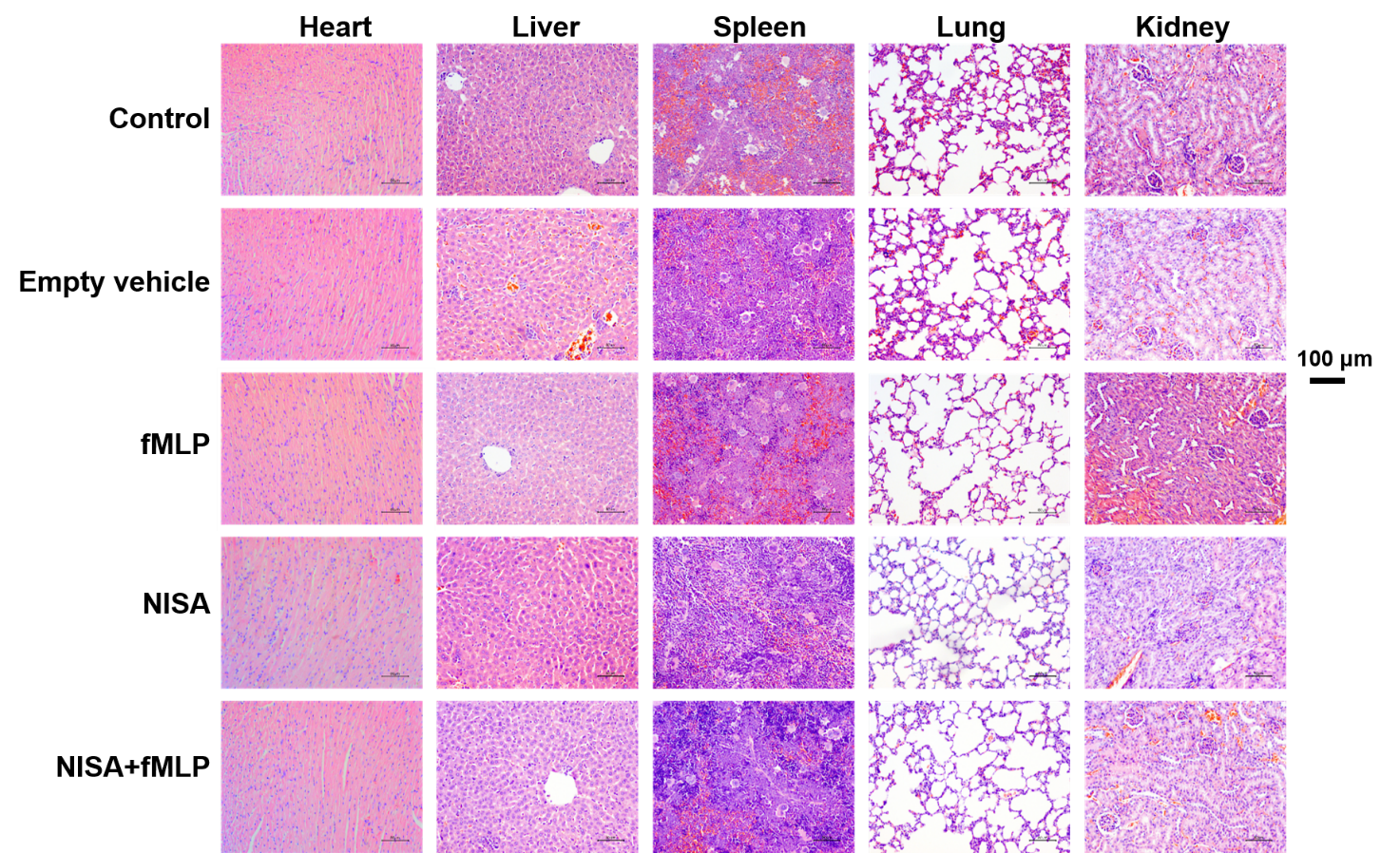


**Figure S15.** On day 9 (24 h after the final injection), 3 mice from each group were sacrificed, and the major organs (heart, liver, spleen, lung, and kidney) of the mice were removed and processed for paraffin sections and histopathological examination (H&E staining).

**
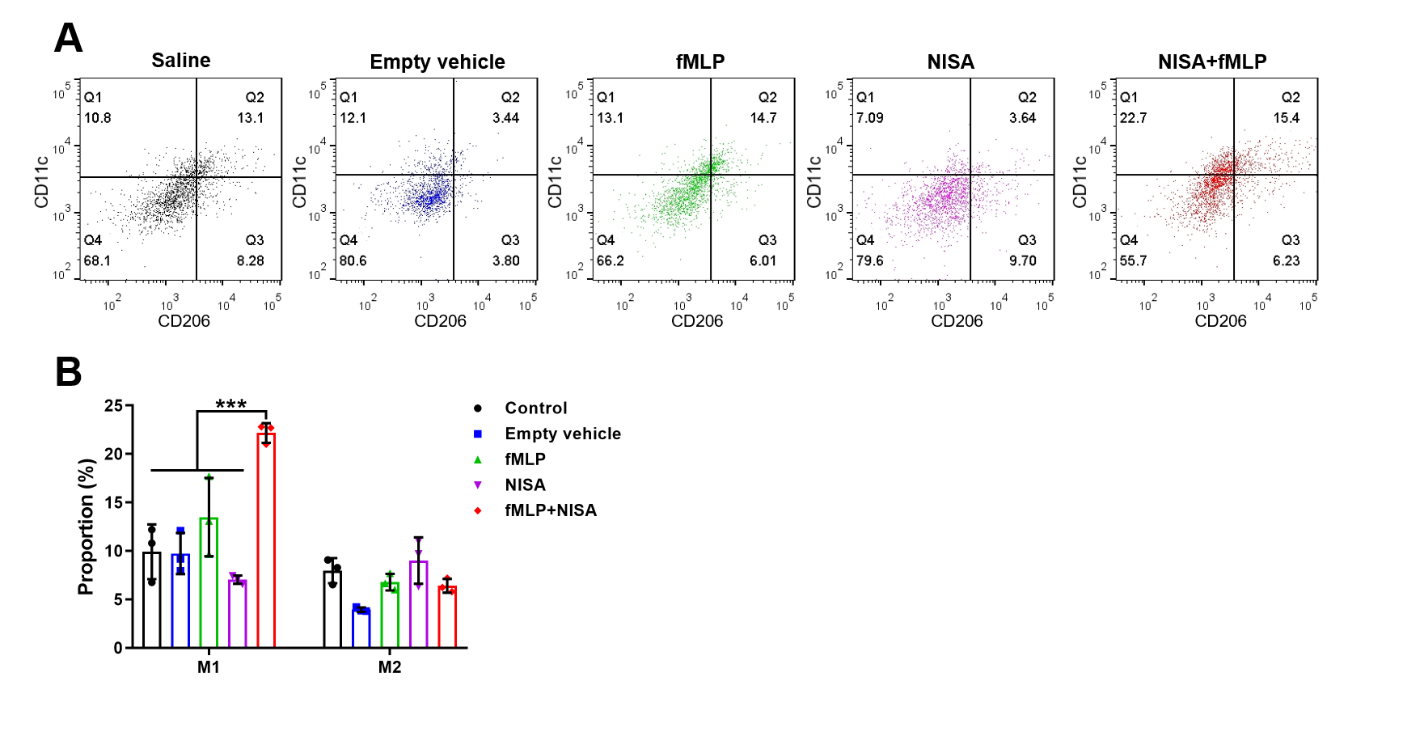
**

**Figure S16.** Macrophage polarization analysis. (**A**) Macrophages are defined as F4/80^+^ cell. M1-like cells are F4/80^+^CD11c^+^CD206^-^, whereas M2-like cells are F4/80^+^CD11c^-^CD206^+^ cells. (**B**) Statistical assay of the M1- and M2-like cell proportion in total macrophages. Data are expressed as mean± SD. n=3. ***p < 0.001.

**
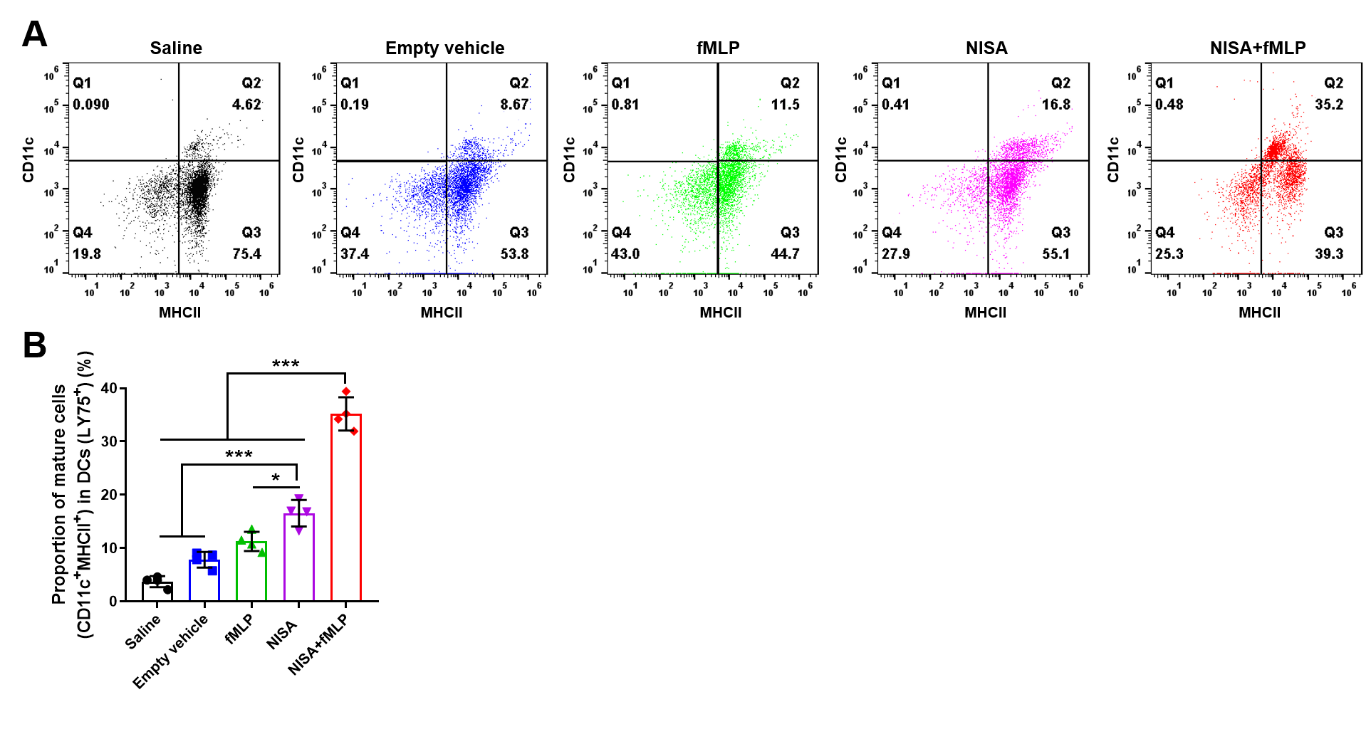
**

**Figure S17.** Analysis of mature DCs in the tumors after various treatments. (**A**) Mature DCs were identified as LY75^+^CD11c^+^MHCII^+^. (**B**) Statistical assay of the mature DC proportions. Data are expressed as mean±SD (n=4) ***p < 0.001.


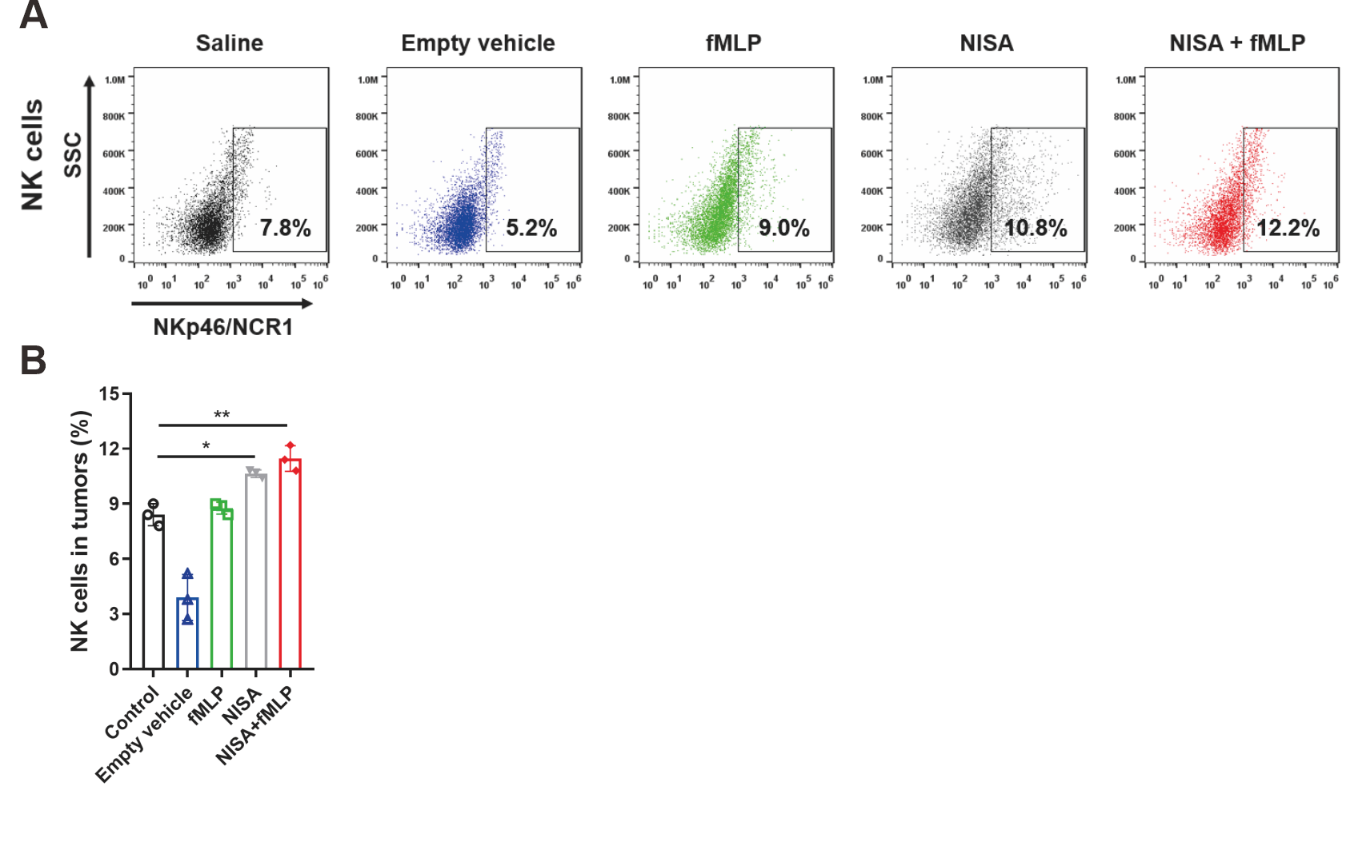


**Figure S18.** Examination of NK cells in tumors after the indicated treatments. (**A**) Representative flow cytometry profiles of NK cells in tumors after the indicated treatment. NK cells were marked with goat anti-mouse NKp46/NCR1 antibody (FITC) (R&D, Minneapolis, MN). (**B**) Statistical assay of the NK cell percentage. Data are expressed as mean ± SD. n=3. *p < 0.05, **p < 0.01.

**Table S1.** Survival analysis of the mice with various treatments.

|  | Median survival (day) | *ILS* (day) ^a^ | % *ILS* ^b^ |
| --- | --- | --- | --- |
| Saline | 26 | - | - |
| Empty vehicle | 30 | 4 | 15.4 |
| fMLP | 35 | 9 | 34.6 |
| NISA | 40 | 14 | 53.8 |
| NISA + fMLP | 44 | 18 | 69.2 |

^a^ *ILS*: Increase in Life Span (T - C), where T and C are the mean survival time of treated mice and control mice from the saline group.

^b^ %*ILS* = (T/C-1)×100%
